# The risks of malariainfection in Kenya in 2009

**DOI:** 10.1186/1471-2334-9-180

**Published:** 2009-11-20

**Authors:** Abdisalan M Noor, Peter W Gething, Victor A Alegana, Anand P Patil, Simon I Hay, Eric Muchiri, Elizabeth Juma, Robert W Snow

**Affiliations:** 1 Malaria Public Health and Epidemiology Group, Centre for Geographic Medicine, KEMRI - University of Oxford - Wellcome Trust Collaborative Programme, Kenyatta National Hospital Grounds (behind NASCOP), P.O. Box 43640-00100, Nairobi, Kenya; 2 Centre for Tropical Medicine, Nuffield Department of Clinical Medicine, University of Oxford, CCVTM, Oxford OX3 7LJ, UK; 3 Spatial Ecology and Epidemiology Group, Tinbergen Building, Department of Zoology, University of Oxford, South Parks Road, Oxford, OX1 3PS, UK; 4 Division of Vector-Borne and Neglected Diseases, Ministry of Public Health and Sanitation, P.O Box 20750, 00100 GPO, Nairobi, Kenya; 5 Division of Malaria Control, Ministry of Public Health and Sanitation, P.O Box 19982, 00202 KNH, Nairobi, Kenya

## Abstract

**Background:**

To design an effective strategy for the control of malaria requires a map of infection and disease risks to select appropriate suites of interventions. Advances in model based geo-statistics and malaria parasite prevalence data assemblies provide unique opportunities to redefine national *Plasmodium falciparum *risk distributions. Here we present a new map of malaria risk for Kenya in 2009.

**Methods:**

*Plasmodium falciparum *parasite rate data were assembled from cross-sectional community based surveys undertaken from 1975 to 2009. Details recorded for each survey included the month and year of the survey, sample size, positivity and the age ranges of sampled population. Data were corrected to a standard age-range of two to less than 10 years (*Pf*PR_2-10_) and each survey location was geo-positioned using national and on-line digital settlement maps. Ecological and climate covariates were matched to each *Pf*PR_2-10 _survey location and examined separately and in combination for relationships to *Pf*PR_2-10_. Significant covariates were then included in a Bayesian geostatistical spatial-temporal framework to predict continuous and categorical maps of mean *Pf*PR_2-10 _at a 1 × 1 km resolution across Kenya for the year 2009. Model hold-out data were used to test the predictive accuracy of the mapped surfaces and distributions of the posterior uncertainty were mapped.

**Results:**

A total of 2,682 estimates of *Pf*PR_2-10 _from surveys undertaken at 2,095 sites between 1975 and 2009 were selected for inclusion in the geo-statistical modeling. The covariates selected for prediction were urbanization; maximum temperature; precipitation; enhanced vegetation index; and distance to main water bodies. The final Bayesian geo-statistical model had a high predictive accuracy with mean error of -0.15% *Pf*PR_2-10_; mean absolute error of 0.38% *Pf*PR_2-10_; and linear correlation between observed and predicted *Pf*PR_2-10 _of 0.81. The majority of Kenya's 2009 population (35.2 million, 86.3%) reside in areas where predicted *Pf*PR_2-10 _is less than 5%; conversely in 2009 only 4.3 million people (10.6%) lived in areas where *Pf*PR_2-10 _was predicted to be ≥40% and were largely located around the shores of Lake Victoria.

**Conclusion:**

Model based geo-statistical methods can be used to interpolate malaria risks in Kenya with precision and our model shows that the majority of Kenyans live in areas of very low *P. falciparum *risk. As malaria interventions go to scale effectively tracking epidemiological changes of risk demands a rigorous effort to document infection prevalence in time and space to remodel risks and redefine intervention priorities over the next 10-15 years.

## Background

As most endemic countries begin to re-focus their malaria control goals, including in some cases a target of elimination [1], contemporary maps that reliably define sub-national variation in disease risk are required to inform priority setting and the selection of appropriate suites of intervention. Recent efforts at developing empirical global maps of *Plasmodium falciparum *risk herald a new era of using maps to define regional populations at risk of malaria to guide the future global malaria control agenda [2]. However, the applicability of malaria risk mapping to make predictions at spatial scales and time points necessary for effective health service planning and review depends largely on the amount and resolution of information available. For example, it is recognized that continental risk maps may not provide sufficient detail and precision for national and sub-national level control priority setting [2].

Kenya is one of very few countries that have a plethora of malaria risk data, spanning over 30 years. The earliest attempts to describe the spatial distribution of malaria risk in Kenya were based on expert opinion of malaria seasons and climate [3]. Between 1998 and 2005 several attempts were made by our group to model the predictive accuracy of this historical map [4,5] or use historical parasite prevalence data and remotely sensed proximates of climate to predict risk using sub-optimal spatial methods that were unable to define fully the uncertainty in the modeled maps [6,7]. Here we present a more robust Bayesian model-based geo-statistical spatial-temporal method to predict the risk of malaria in Kenya in 2009 using the largest assembled contemporary empirical evidence for any country in Africa. As a new phase of malaria control in Kenya begins, the implications of the resulting malaria risk map for decision makers and the prospects for the future of malaria control nationwide are discussed.

## Methods

### *P. falciparum *parasite rate as a marker of risk

There are many measures of the intensity of malaria transmission [8,9]. Direct measures of transmission intensity applicable for malaria modeling include the entomological inoculation rate (EIR) and the basic reproductive number (*R*_*o*_). EIR is the number of parasite-specific infectious bites received by a person per unit time and *R*_*o *_is the average number of secondary infections resulting from one infected individual being introduced into a non-immune host population. These indices are rarely measured, however, thus limiting their utility for spatial modeling [10]. An alternative measure of *P. falciparum *malaria risk is the parasite rate (*Pf*PR), which is the proportion of a random sample of population with malaria parasites in their peripheral blood, used frequently to define transmission intensity since the 1950's [11] and has a predictable mathematical relationship to the rarely sampled measures of EIR and *R*_*o *_[12-14]. The *Pf*PR has therefore become the benchmark indicator by which malaria risk is modeled and mapped in Africa [2,6,7,15-17].

### Data identification

*Pf*PR survey data were identified using basic search principles and inclusion criteria described elsewhere [18] with two notable exceptions: firstly survey data were included if surveys were undertaken from 1^st ^January 1975, because of the rich Ministry of Health survey data between 1975 and 1984 [19]; and secondly no restriction was placed on sample size for inclusion in the spatial modeling [2].

Data searches included online searches for peer-reviewed publications using PubMed [20] and African e-repositories [21]; manual searches of monthly returns archived from over 40 field stations maintained by the Ministry of Health's Division of Vector Borne Diseases; reviews of master's and doctoral thesis titles and abstracts from the Universities of Nairobi and Jomo Kenyatta; access to household survey data supported as part of national [22] or sub-national sample surveys on malaria or nutrition [23,24]; and an extensive correspondence and data sharing exercise with the prolific malaria research community in Kenya or those working in the country but based overseas [25]. Data searches began in 2005 and were completed with final reviews of published reports and correspondence with national research groups on 31^st ^March 2009. All data were entered into a customized Microsoft Access (Microsoft 2007) database to include information on survey location, survey timing (month and year), age ranges of the sampled population, sample size, numbers reported positive for *P. falciparum *infection and the methods of parasite detection [18].

### Pre-processing of *PfPR *survey data

#### Geo-location techniques

A series of independent databases of cities, towns and villages developed since 2004 with longitude and latitude coordinates from Global Positioning System (GPS) recordings are available in Kenya. These include a national schools database developed through a mapping project in 2008 by the Ministry of Education [26]; a database of settlements connected to the classified motorable road network compiled as part of a road mapping project by the Ministry of Roads and Public Works [27]; and a variety of smaller databases developed as part of research projects or development programmes. In addition, a database of villages digitised from topographical maps in 2002 was obtained from the International Livestock Research Institute. These databases were first used to geo-position survey locations with priority given to the GPS sources. Where survey locations could not be geo-positioned from any of these national databases, digital databases such Microsoft Encarta (Microsoft 2007), Alexandria Digital Library [28] and Falling Rain Genomics Inc. Global Gazetteer [29] were used. A database of enumeration areas for the 1999 census obtained from the Kenya National Bureau of Statistics was used as a final source if survey data could not be positioned using the other sources. Survey locations were classified as points if they could be positioned to an area ≤10 km^2^; wide area (>10 km^2 ^to <25 km^2^); or polygon (≥25 km^2^) [18].

#### Age standardization of PfPR

Under stable endemic transmission *Pf*PR is age-dependent and rises during early childhood, peaks in older children and falls through adolescence and adulthood, the rate of decline a consequence of development of anti-parasitic immunity [14]. *Pf*PR surveys, however, are often reported for a variety of age-ranges. The assembled *Pf*PR data were therefore standardized to the classical age-range of 2 to less than 10 years using an algorithm based on catalytic conversion models first adapted for malaria by Pull and Grab [30] and modified by Smith *et al.*, [14]. This age-standardized parasite rate, henceforth referred to as *Pf*PR_2-10_, was computed for each survey report [2].

### Assembling and testing ecological and climate covariates

A number of ecological and climatic factors affect the development and survival of the *P. falciparum *parasite and the malaria-transmitting *Anopheles *vector [31]. National and regional malaria risk modelling efforts have used various proximate determinants of infection and disease risk including continuous and categorical forms of urbanization [17,32,33], rainfall [4,15-17,34-37], vegetation coverage [15,17,32,35,38,39], aridity [36,40], distance to water bodies [5,15-17,35-37,41,42], altitude [4,5,36,37] and temperature [4,5,15-17,34-37,39,41]. We elected to explore the discriminatory effects of these covariates derived from census, meteorological, topographical and remotely sensed satellite sources all described in detail in Additional File 1. All covariates were re-sampled to 1 × 1 km spatial resolutions using ArcGIS 9.2 (ESRI, Redlands, CA, USA) and matched to survey locations where the numbers of individuals examined were ≥50 (n = 2,094).

The relationships of the covariates in their continuous and categorical forms were first visually examined against *Pf*PR_2-10 _data using scatter and box plots. These were used to aggregate the covariates into suitable categories that corresponded to biologically appropriate definitions, previous applications of remotely sensed variables and retention of effective sample sizes (see Additional File 1). A univariate non-spatial binomial logistic regression model was then implemented for each covariate with *Pf*PR_2-10 _as the dependent variable in Stata/SE Version 10 (Stata Corporation, College Station, TX, USA). The results of the univariate analyses were used to determine the relative strength of each candidate covariate as a predictor of *Pf*PR_2-10 _and identify those which qualified for inclusion in the Bayesian geostatistical model. First, where there was more than one plausible way of categorizing a covariate, the size of the odds ratio, the Wald's p-value and the value of Akaike Information Criterion (AIC), a measure of the goodness of fit of an estimated statistical model [35], were used to determine which approach resulted in categories with the strongest association with *Pf*PR_2-10 _[SI 1]. Once the best categorizations were determined, a collinearity test of all the covariates was undertaken and if a pair had a correlation coefficient > 0.9 [43], the variable with the highest value of AIC was dropped from subsequent analysis. The selected covariates were then analysed in a binomial multivariate logistic regression with *Pf*PR_2-10 _as the dependent variable. Using backwards variable elimination, covariates with Wald's P > 0.2 were removed step-wise until a fully reduced model was achieved.

### Bayesian space-time models

Using the Kenya *Pf*PR_2-10 _data and the selected covariates, a spatial-temporal Bayesian generalized linear geostatistical model [2] was implemented to predict a malaria map of Kenya for 2009. Bayesian geostatistical models provide the ability to predict values of a spatially continuous event at unsampled locations using combinations of the sampled data in space and time, and importantly allow for calculation of robust uncertainty estimates around model predictions [2,43,44]. The underlying assumption of the Kenya *Pf*PR_2-10 _model was that the probability of prevalence at any survey location was the product of two factors. First, a continuous function of the time and location of the survey, modified by a set of covariates, and modelled as a transformation of a space-time Gaussian random field. Second, a factor depending on the age range of individuals sampled in each survey. The distribution of the second factor [2] was based on the procedure described by Smith *et al. *[14]. The Bayesian spatial-temporal model was implemented in two parts starting with an inference stage in which a Markov Chain Monte Carlo (MCMC) algorithm was used to generate samples from the joint posterior distribution of the parameter set and the space-time random field at the data locations. This was followed by a prediction stage in which samples were generated from the posterior distribution of *Pf*PR_2-10 _at each prediction location on a 1 × 1 km grid. Details of the spatial-temporal Bayesian geostatistical models are presented in Additional File 2.

### Model validation and measures of uncertainty

#### Selection of model validation test data

To ensure that the validation data were spatially representative of the whole country, a spatial declustering algorithm [2] was implemented. This algorithm defined Thiessen polygons whose boundaries enclosed the area that was closest to each point relative to all other points around each survey location. A 10% sample of the larger Kenya *Pf*PR_2-10 _dataset was then drawn randomly. Each data point had a probability of selection proportional to the area of its Thiessen polygon so that data located in densely surveyed regions had a lower probability of selection than those in sparsely surveyed regions [45]. The Bayesian spatio-temporal geostatistical model was then implemented in full using the remaining 90% of data.

#### Computing model accuracy and uncertainty

A series of validation statistics were computed by comparing the predicted *Pf*PR_2-10 _values to actual *Pf*PR_2-10 _observed at the validation locations. The validation statistics were: the linear correlation coefficient; mean error (ME); and mean absolute error (MAE) is a measure of the bias of predictions (the overall tendency to over or under predict). Finally, the probability of membership of a survey location to its assigned endemicity class (see next section) was computed as a measure of uncertainty. These probabilities, ranging from 1 (no uncertainty in class membership) to 0.14 (membership equally likely to all classes) were computed from the posterior distributions resulting from the Bayesian geostatistical model as explained in detail in Additional File 2.

### Malaria risk classifications and estimations of populations exposed to risk

Seven endemicity classes of *Pf*PR_2-10 _were selected: <0.1%; ≥0.1% and < 1%; ≥1% and <5%; ≥5% and <10%; ≥10% and <20%; ≥20% and <40%; ≥40%. These classes were selected as they can be used to compute approximates of the traditional measures of endemicity [11], are congruent with recommendations for the selection of suites of vector control and the timelines to effective transmission control [9,46,47] and allow for interpretation of lower risk categories where the predominant spatial risks are not among the higher endemicity classes. The probability of membership to each endemicity class was estimated from the posterior probability distributions of *Pf*PR_2-10 _for each pixel generated by the Bayesian geostatistical model, as described in Additional File 2.

A high-resolution (100 × 100 m) population distribution map of Kenya [48] was used to compute the number of people in each of the malaria endemicity classes. This map was constructed from a combination of satellite imagery and land cover maps which were used to develop models that identified the location of settlements [48,49]. The modelled settlements map was then used to redistribute census population counts within the small enumeration area polygons. The resulting high-resolution map represented estimated population distribution in Kenya for the year 2000. This raster population surface was then projected to 2009 using provincial inter-censal growth rates from the 1999 national census [50]. The raster malaria endemicity map was then overlaid on the projected population map and the number of people in each endemicity class, overall and by province, was extracted using ArcGIS 9.2 *Spatial Analyst *tool.

## Results

### Assembled data

**Figure 1:**
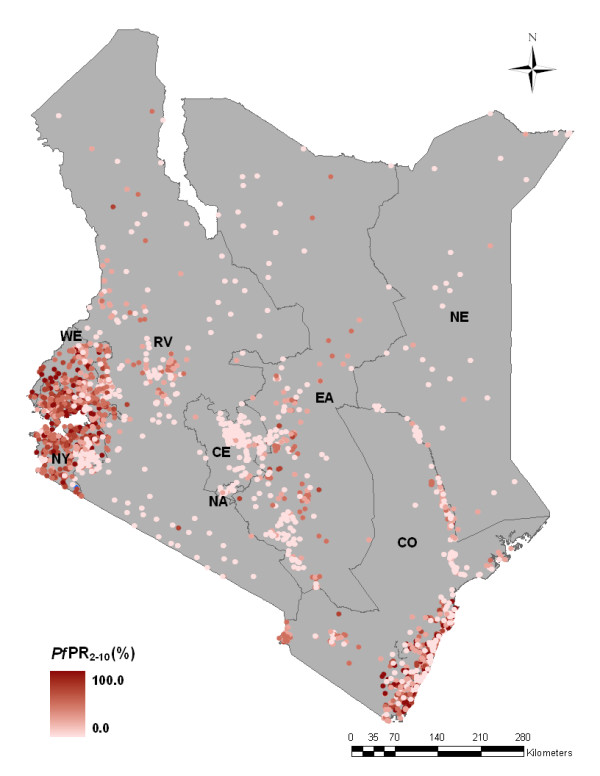
**Province map of Kenya showing the distribution of 2,095 spatially unique survey locations out of the 2,682 selected for analysis**. Colours ranging from light pink to dark red represent increasing *Pf*PR_2-10_. Where there were repeat surveys at the same location (n = 587), *Pf*PR_2-10 _data are displayed from the most recent survey. CE = Central province; CO = Coast province; EA = Eastern province; NA = Nairobi province; NE = North Eastern province; NY = Nyanza province; RV = Rift Valley province; and WE = Western province.

**Figure 2:**
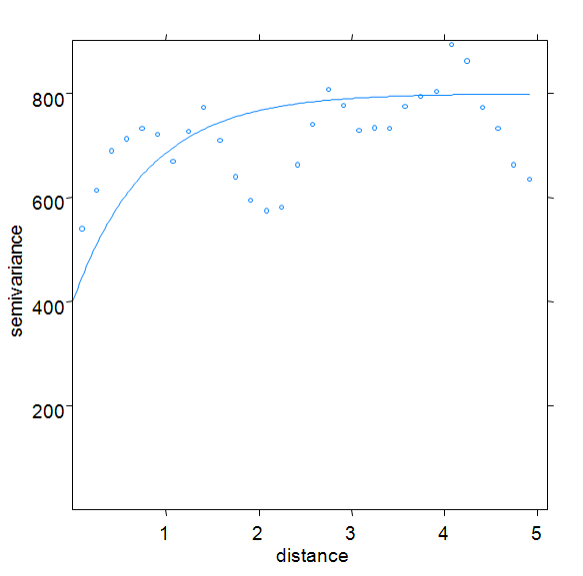
**Sample semi-variograms of *Pf*PR_2-10 _dataset (n = 2,682) indicating the presence of spatial autocorrelation in the *Pf*PR_2-10 _data up to lags of 1 decimal degree or the equivalent of ~111 km at the equator**.

A total of 2,756 *Pf*PR random sample surveys were assembled for the period 1975-2009. Of these, 74 survey locations were excluded from analysis because they were polygons (n = 30); were not positioned (n = 41); or were missing survey month (n = 3). Of the remaining 2,682 data points (Table 1), 1,672 (62.3%) were obtained from Ministry of Health reports; 364 (13.6%) from peer-reviewed journal articles and conference abstracts; 111 (4.1%) from theses; and 535 (19.9%) from unpublished grey literature and personal communication sources. The majority of surveys were undertaken in rural areas (n = 2,153, 80.3%). A significant number of surveys were undertaken as part of school health surveillance since 1975 (n = 1,372, 51.1%). Of the total survey sample 2,095 (78.1%) were spatially unique locations (shown in Figure 1) while the remainder (587, 21.9%) were surveys undertaken in the same locations but at different times between 1975 and 2009. Most of the survey locations (73%) were positioned using GPS coordinates. A sample semivariogram of the *Pf*PR_2-10 _data indicated the presence of spatial autocorrelation up to lags of 1 decimal degree or the equivalent of ~111 km at the equator (Figure 2).

**Table 1:** Summary of the Kenya *Pf*PR survey data showing the number of survey data points and the sample size across different categories.

	Survey data points*	Spatially unique data points	Survey locations with no positive *P. falciparum *samples	Persons examined
**Residence**				
Rural	2,133	1,706	321	334,993
Urban	549	389	87	52,799

**Upper age (years) sampled**				
≤5	449	410	161	20,570
>5 and ≤10	364	255	12	66,893
>10 and ≤20	1,596	1,209	224	247,616
>20	273	221	11	52,713

**Decade**				
1975-1984	698	499	124	119,181
1985-1994	611	476	14	135,799
1995-2004	610	452	43	69,899
2005-2009	763	668	227	62,913

**Province**				
Central	140	134	106	13,225
Coast	872	557	90	108,335
Eastern	233	210	78	31,186
Nairobi	15	15	2	3,105
North Eastern	56	54	30	5,437
Nyanza	729	605	23	127,383
Rift Valley	365	301	70	56,880
Western	272	219	9	42,241

**Type of sample survey**				
Community	1,310	993	177	179,939
School	1,372	1,102	231	207,853

**Method of malaria testing**				
Microscopy	2,107	1,549	177	353,449
Rapid diagnostic test	575	546	231	34,343

**Total**	**2,682**	**2,095**	**408**	**387,792**

*Data from 16 survey locations for which *Pf*PR values were available but the number examined and number positive were not are not included in Table 1. Survey data points presented here include 2,095 spatially unique and 587 spatial duplicates but temporally unique points.

### Testing of climate and ecological covariates

The univariate analysis showed that all the biologically selected categorical covariates were statistically significant predictors of differences in *Pf*PR_2-10 _(see Additional File 1 and Table 2). There was reduced risk of infection in areas that were: urban compared to rural; of minimum average annual temperature of <16°C compared to ≥16°C; of maximum average annual temperatures of <25°C or >30°C compared to between 25°C - 30°C; of zero or 1-3 sets of three continuous months of precipitation >60 mm in an average year compared to corresponding precipitation patterns that occurred >3 sets in an average year; where EVI was ≤0.3 compared to >0.3; where the survey was located at an altitude of ≤500 m or >1500 m compared to between >500-1500 m above sea level; and were at a distance to main water bodies of >12 km relative to ≤12 km (see Additional File 1 and Table 2).

**Table 2:** Results of univariate and multivariate analysis of the ecological and climatic covariates (SI 1) against Kenya *Pf*PR_2-10 _data of sample size ≥50 individuals.

	*Pf*PR_2-10_			
	
	Number of survey locations	Mean (median) PfPR_2-10_, Chi^2 ^(P-value)	Univariate regression*: Odds Ratio (95% CI), P-value	Multivariate regression*: Odds Ratio (95% CI), P-value
**Categorical covariates**				

**Urban**				
Rural	1636	27.6 (21.9)	Ref	Ref
Urban	458	15.4 (11.9)	0.48 (0.34, 0.66), <0.001	0.50 (0.36, 0.70), <0.001
		3300, <0.001		

**Maximum temperature (Degrees Celsius)**				
≤25	214	7.7 (2.3)	0.20 (0.10, 0.41), <0.001	0.25 (0.12, 0.52), <0.001
25-30	1628	29.8 (25.9)	Ref	Ref
>30	252	16.2 (9.2)	0.46 (0.35, 0.60), <0.001	0.61 (0.44, 0.85), 0.003
		4700.0, <0.001		

**Minimum temperature (Degrees Celsius)**				
<16	928	23.5 (17.1)	0.81 (0.66, 0.97), <0.036	
≥16	1166	27.6 (21.9)	Ref	
		754.7, <0.001		

**Sets of 3 consecutive months in an average year with precipitation >60 mm**				
0	1398	13.7 (9.0)	0.37 (0.26, 0.51), <0.001	0.53 (0.35, 0.83), 0.005
1-3	333	19.6 (12.4)	0.56 (0.42, 0.74), <0.001	0.63 (0.46, 0.85), 0.003
>3	363	30.3 (26.1)	Ref	Ref
		8200.0, <0.001		

**Enhanced vegetation index**				
> 0.3	1534	16.9 (11.3)	Ref	Ref
≤0.3	560	29.0 (24.4)	0.50 (0.39, 0.64), <0.001	0.78 (0.57, 1.06), 0.114
		3300.1, <0.001		

**Altitude (m)**				
0-500	689	22.2 (13.0)	0.59 (0.47, 0.74), <0.001	
>500-1500	860	32.6 (29.1)	Ref	
>1500	545	19.4 (13.0)	0.50 (0.39, 0.64), <0.001	
		4100.2, <0.001		

**Distance (km) to main water bodies**				
≤12 mean distance	1306	28.6 (23.5)	Ref	
>12 mean distance	788	21.1 (14.5)	0.67 (0.54, 0.82), <0.001	0.62(0.49, 0.77), <0.001
		3300, <0.001		

*The category with the highest median *Pf*PR_2-10 _is used as the reference class. Therefore the odds ratios are expected to be below 1.00 for all covariates.

In the multivariate regression, however, only the classifications of urban-rural; maximum temperature; precipitation; EVI and distance to main water bodies were included (Table 2). Altitude and minimum temperature were excluded from the multivariate analysis because they were highly correlated with each other (R^2 ^= 0.97) and with maximum temperature (R^2 ^> 0.70) and both had comparatively higher AIC values [SI 1]. From the multivariate analysis the risk of malaria parasite infection was lower in locations that were: urban compared to rural (odds ratio, 95% CI: 0.50, 0.36-0.70, p < 0.001); of maximum temperatures <25°C (0.25, 0.12-0.52, p < 0.001) or >30°C (0.61, 0.44-085, p = 0.003) compared to between 25°C- 30°C; of zero (0.53, 0.36-0.83, p = 0.005) or 1-3 (0.63, 0.46-0.85, p = 0.003) sets of three continuous months of precipitation >60 mm in average year compared to >3 sets; and at distance to water bodies of >12 km (0.62, 0.49-0.77, p < 0.001) relative to ≤12 km (Table 2). Although there was a reduced risk of infection prevalence at EVI of ≤0.3 (0.77, 0.57-1.06) compared to >0.3, this was not statistically significant (p = 0.114). This, however, did not preclude the inclusion of EVI in the final model set as it still met the inclusion criteria with a P value < 0.2 and the AIC value of the multivariate model was lower with it compared to without.

### Bayesian predicted risk projected to 2009

**Figure 3:**
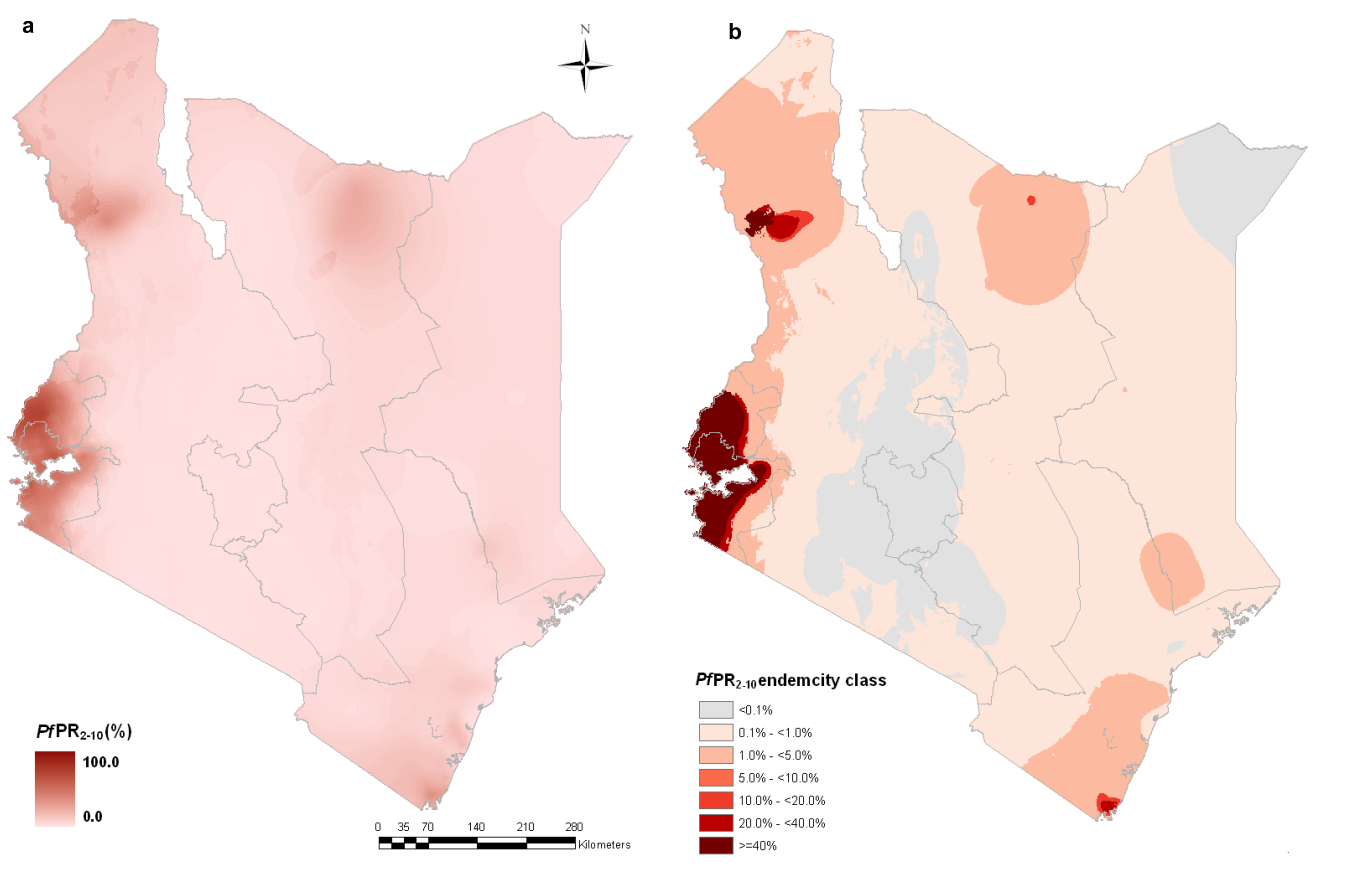
**Spatial distribution of *P. falciparum *malaria in Kenya at 1×1 km spatial resolution**. **a) **continuous posterior mean *Pf*PR_2-10 _prediction; **b) **endemicity classes: *Pf*PR_2-10 _< 0.1%; ≥0.1 and < 1%; ≥1 and <5%; ≥5 and <10%; ≥10 and <20%; ≥20 and <40%; ≥40%.

The 2009 map of the predicted posterior mean distribution of *Pf*PR_2-10 _is shown in Figure 3a. The predicted malaria endemicity class map is shown in Figure 3b and indicates that the majority of the country's surface area falls into endemicity classes of <5% *Pf*PR_2-10_. The lowest endemicity class (< 0.1% *Pf*PR_2-10_) covers most of Nairobi and Central provinces and some parts of the Eastern and Rift Valley provinces (Figure 3b). The endemicity class of between 0.1 and 1% covers most of the North Eastern, Eastern, Rift Valley and Coast provinces. High transmission areas (endemicity class ≥40% *Pf*PR_2-10_) were predicted mainly in small parts of Nyanza province along the shores of Lake Victoria and cover <2% of the total area of Kenya.

### Model validation

**Figure 4:**
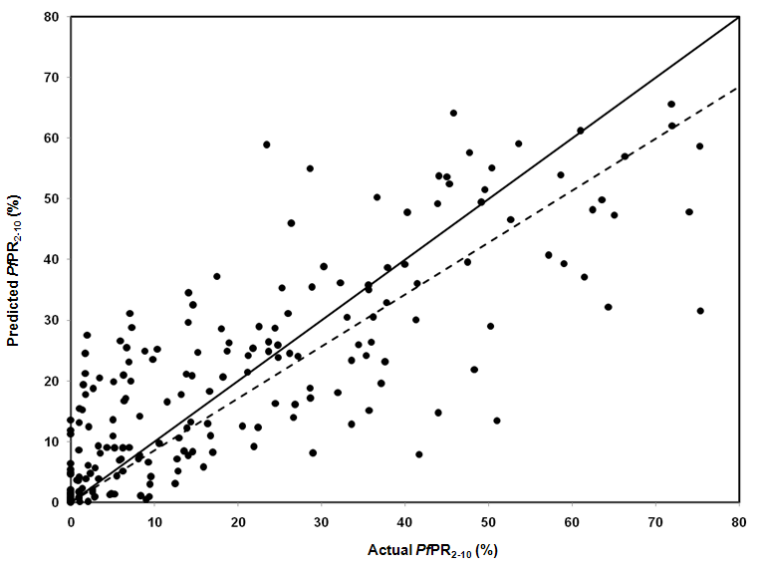
**Scatter plot of actual versus predicted point-values of *Pf*PR_2-10 _for the selection validation set (n = 210)**. The linear correlation (R) of the actual versus predicted *Pf*PR_2-10 _was 0.81. The solid black line shows the line of perfect fit; the dashed black line is the trend line with intercept set at zero.

**Figure 5:**
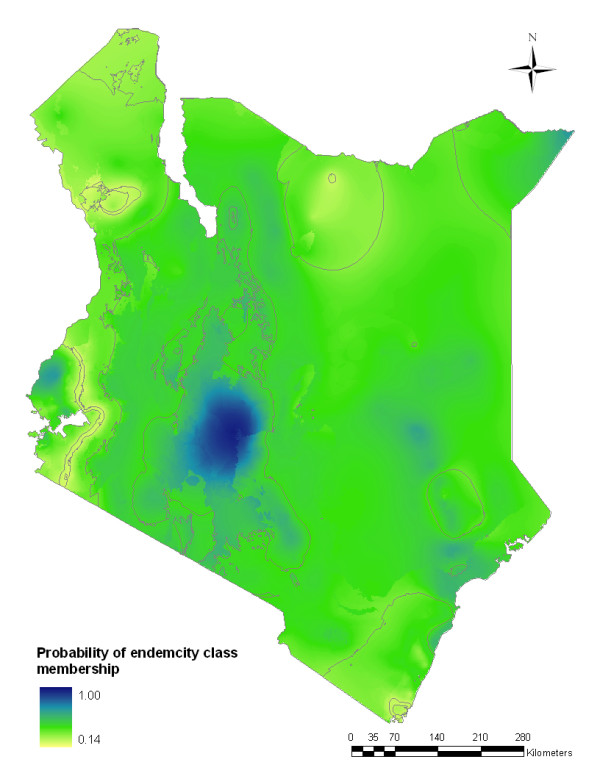
**Spatial distribution of probability of membership of *P. falciparum *malaria endemicity class in Kenya at 1 × 1 km spatial resolution**. Given that there are seven endemicity classes, the lowest probability of class assignment is 0.14. Any value above 0.14 is better than a chance allocation to the endemicity class. Lines shown on the map represent the contours of the different endemicity classes shown in Figure 3.

The mean error in the prediction of *Pf*PR_2-10 _for 2009 revealed low overall bias with a slight tendency to under-estimate predictions by -0.15% (Table 3). The mean absolute error also showed a relatively moderate model precision with low average error of predictions of 0.38%. The correlation between the actual and predicted values for the hold-out set was 0.81 indicating a strong linear agreement (Figure 4). In assessing the endemicity classes, the overall probabilities of membership of the predicted class were all greater than the chance assignment value of 0.14 and in most of the country was greater than 0.45 (Figure 5).

**Table 3:** Summary of validation statistics for predicting continuous *Pf*PR_2-10 _in Kenya based on a validation set of 210 data points.

Validation measure	
Linear correlation coefficient of predicted versus observed	0.81
Mean error (% *Pf*PR_2-10_)	-0.15
Mean absolute error (% *Pf*PR_2-10_)	0.38

Mean error is a measure of the bias of predictions (the overall tendency to over or under predict) whilst mean absolute error is a measure of overall precision (the average magnitude of error in individual predictions).

### Population at risk in 2009

Of the estimated 40.8 million people in Kenya in 2009, the majority (65.6%) lived in areas where malaria risk was <1% *Pf*PR_2-10 _with 14.8 million (36.2%) living in areas of < 0.1% *Pf*PR_2-10 _(Table 4). Approximately 8.5 million (20.8%) people lived in areas where transmission risks were predicted to be between 1% and 5% *Pf*PR_2-10 _in 2009; while the remaining population (5.6 million, 13.7%) lived in areas of risk ≥5% *Pf*PR_2-10_, of which 4.3 million (10.6% of Kenya's 2009 population) were predicted to be living in the highest transmission areas of ≥40% *Pf*PR_2-10 _(Table 4).

**Table 4:** Total population (in millions) at different risks of *P. falciparum *transmission in 2009 in Kenya

*Pf*PR_2-10_	< 0.1%	≥ 0.1 - <1.0%	≥ 1.0 - <5.0%	≥ 5.0-10.0%	≥ 10.0 - <20.0%	≥ 20.0 - <40.0%	≥ 40.0
Central	4.30	0.04	0.00	0.00	0.00	0.00	0.00
Coast	0.01	0.96	2.32	0.00	0.07	0.05	0.00
Eastern	1.81	3.73	0.09	0.00	0.00	0.00	0.00
Nairobi	5.51	0.00	0.00	0.00	0.00	0.00	0.00
North Eastern	0.45	1.62	0.07	0.00	0.00	0.00	0.00
Nyanza	0.00	0.43	2.17	0.00	0.01	0.61	2.22
Rift Valley	2.68	5.19	2.13	0.00	0.02	0.01	0.01
Western	0.00	0.00	1.70	0.00	0.03	0.43	2.10

**Total** **(%)**	**14.77** **(36.2)**	**11.98** **(29.4)**	**8.48** **(20.8)**	**0.00** **(0.0)**	**0.13** **(0.003)**	**1.10** **(0.03)**	**4.33** **(10.6)**

Mean error is a measure of the bias of predictions (the overall tendency to over or under predict) whilst mean absolute error is a measure of overall precision (the average magnitude of error in individual predictions).

## Discussion

We have assembled over 2,600 independent, empirical survey estimates of *P. falciparum *infection prevalence in Kenya and used these data to generate a contemporary map of infection prevalence at a 1 × 1 km resolution for the year 2009 using space-time geostatistical models within a Bayesian framework. The modeled distribution had a high predictive accuracy as shown by the low values of ME and MAE and high correlation between predicted and observed *Pf*PR_2-10 _(Table 3 & Figure 4). The probability of endemicity class membership were also generally moderate to high across the country with the exception of small pockets of the low population-density areas of the northern districts where there was relatively sparse distribution of input data in time and space (Figure 5). This mapped distribution of malaria risk represents the most accurate depiction of parasite exposure described for Kenya since efforts to map risk began in the 1950's [2,3,5,6]. More importantly it represents a distribution of risk in 2009 serving as a contemporary basis upon which to design the future of malaria control in Kenya.

The use of a carefully selected suite of covariates to inform the prediction of risk is a departure from the current Malaria Atlas Project approach, with the exception of the use of urban-rural classification [2], but consistent with other approaches to modeling malaria distributions [5,6,15-17,35]. In fact several discrete categories of the covariates were as different in infection prevalence as the differences described for urban versus rural surveys. We elected not to include a mask of zero or unstable transmission based upon temperature and aridity as developed previously by Guerra et al, [40]. Rather we have assumed that these climatic drivers of transmission would be captured within the model and have chosen to bin the risk classes within much smaller *Pf*PR_2-10 _ranges at the lowest end of the transmission spectrum. The lowest risk class encompasses predicted *Pf*PR_2-10 _between 0 and < 0.1% and covers approximately 91,000 km^2 ^within the Nairobi and Central provinces and small parts of Eastern and Rift Valley (Figure 3). Defining absolute zero transmission is conceptually difficult and practically impossible to measure empirically using *Pf*PR_2-10 _and we therefore feel that the more conservative and inclusive approach used here allows for the possibility of transmission until proven otherwise.

What is striking about the contemporary 2009 distribution of malaria infection risk is the enormity of Kenya's land surface under very low intensity transmission. Over 94% of Kenya's surface area is predicted to be exposed to a *Pf*PR_2-10 _of less than 5% and is home to 86% of Kenya's projected population in 2009 (Table 3). Approximately 66% of the 2009 population live in areas where infection prevalence is less than 1%, including a large majority where risks are hard to detect empirically (*Pf*PR_2-10 _< 0.1%) (Table 3). Conversely areas of high transmission, as defined by a *Pf*PR_2-10 _of ≥40%, representing areas expected to be intractable to immediate reductions in parasite prevalence with scaled-up use of insecticide treated nets [47] are located in the strip of land along the shores of Lake Victoria (Figure 3). In 2009 only 11% of Kenya's population was exposed to this highest transmission intensity class (Table 3). Historically holo-to-hyperendemic transmission (≥50% *Pf*PR_2-10_) was thought to exist across much larger reaches of the Kenyan coast, around Lake Victoria and along the Tana River [3,51]. In the present modeled iteration of *Pf*PR_2-10 _holo-endemic transmission (>75% *Pf*PR_2-10_) no longer exists and hyper-endemic transmission is constrained to small pockets within the darkest red class shown in Figure 3. Although this study doesn't present change of infection risk over time, it seems plausible that across much of Kenya the extent and intensity of *P. falciparum *transmission has undergone a recent decline with increasing spatial areas and populations becoming exposed to lower and lower risks of parasite exposure. This has implications for a changing clinical epidemiology in areas undergoing transition, with older children becoming increasingly at risk of severe clinical outcomes [52-55] but more importantly as communities transition to very low levels of parasite exposure overall malaria morbidity and mortality will decline substantially [52,56].

Although the model is characterized by generally low uncertainties, the pockets of greatest predicted uncertainty are located in the northern districts of Turkana, Marsabit and Moyale (Figure 5). Surprisingly, pockets of risk >10% *Pf*PR_2-10 _were observed in these hot and generally arid parts of the country traditionally regarded to be of unstable low risk. These areas, which generally have low population densities and have traditionally not been targeted for scaling of malaria preventive interventions, exhibit highly focal transmission close to water features, such as the Turkwell, Tana and Kerio rivers and were referred to in historical maps as 'malarious near water' [3]. Because of their presumed low risk, few empirical studies of malaria have been undertaken in these areas. The malaria situation among these poor, pastoralist communities remains ill-defined. In addition there are some important methodological constraints to defining risk in areas of very low transmission and new approaches to micro-geographic Bayesian modeling of risk based upon a presence/absence criterion may be required to improve risk mapping in these areas where the majority risk is negligible, seasonal and exceptionally heterogeneous, associated with the presence of water features.

Further improvements in malaria risk mapping using *Pf*PR_2-10 _might be achieved if the prediction models were corrected for whether microscopy or RDT was used to examine parasitaemia given the varying sensitivities and specificities of the two methods [57]. In this study, however, this was not possible because information on the type of RDT used and the quality of microscopy was lacking for most surveys. In future, it may be feasible to develop universal models that correct for sensitivity/specificity differentials of the methods used to test for infection, preferably based on large-scale population surveys which have used both RDT and microscopy for the same individuals with the appropriate quality assurance and external validity.

The prospects for Kenya to transition the majority of its population living in high transmission areas in the next 10 years to areas of low (*Pf*PR_2-10 _<5%) or very low endemicity (*Pf*PR_2-10 _<1%) look promising. It is however important to emphasize the control implications of this low stable endemic control. There appear to be areas along the Kenyan coast that currently experience risks associated with a *Pf*PR_2-10 _<5% and are likely to have transitioned to this state from meso-hyperendemic conditions. If this has been achieved through the scaled-up use of insecticide treated nets (ITN) then universal coverage must be maintained as withdrawal of ITN would result in a devastating rebound where vectors persist but functional immunity has been modified among the human host population. Conversely in areas that have historically had low or very low transmission, for example in semi-arid areas, the adoption of ITN may not be the most cost-effective strategy. As such all areas of similar contemporary risk may not be equivalent in terms of strategic control. One therefore must interpret contemporary distributions of risk for control planning in concert with the potential vulnerabilities of transmission based upon vector distributions or historical descriptions of risk. For Kenya it is also important to recognize that there are vast areas where infection risks are low and have historically been low because of their ecological niches (arid, urban or at high elevation). While these communities enjoy a low risk of infection, risks are not absent and thus cost-efficient suites of interventions must be tailored to meet their needs. This poses a challenge where universal coverage of ITN and presumptive fever treatment with Artemisinin based combination therapy remain the single bedrock of most national malaria control strategies across Africa.

## Conclusion

There remains some debate over the feasibility of malaria elimination in Africa [58-61]. Kenya is an example where infection prevalence is low across large parts of the country. However moderate-to-high risks remain in well defined areas, some of which share borders with neighboring countries and risks are not absent from marginalized hard-to-reach communities in semi-arid areas of the country. Successes in reducing infection prevalence in some areas [[55], Okiro EA, Alegana VA, Noor AM, Mutheu JJ, Juma E, Snow RW: Malaria paediatric hospitalization between 1999 and 2008 across Kenya. Submitted] that have led to reductions in disease burden [[54,55], Okiro EA, Alegana VA, Noor AM, Mutheu JJ, Juma E, Snow RW: Malaria paediatric hospitalization between 1999 and 2008 across Kenya. Submitted] must be maintained and expanded and not viewed as 'job finished'. This alone may pose challenges for sustained financing. What is encouraging is that risks can be measured using survey data of infection prevalence; we have shown here that their spatial distribution can be modeled and mapped with accuracy; and that this can become the basis for judging the future success of control nationwide using data that does not depend upon opportunistic historical surveys. To this end the Kenyan Ministry of Health proposes to maintain annual surveys of malaria infection prevalence among school children as part of its monitoring of the revised national malaria strategy 2009-2017 (E Juma, personal communication). This will represent the first attempt in Africa to serially measure, map and model changing endemicity as part of scaled intervention coverage and where robust baseline endemicity for 2009 exists to judge success.

## Abbreviations

EA: Enumeration area; EVI: Enhanced Vegetation Index; GPS: Global Positioning System; ILRI: International Livestock Research Institute; KNBS: Kenya National Bureau of Statistics; MAE: Mean Absolute Error; MCMC: Markov Chain Monte Carlo; ME: Mean Error; NASA: National Aeronautics and Space Administration; NDVI: Normalized Difference Vegetation Index; NGA: National Geospatial-Intelligence Agency; *Pf*PR: *P. falciparum *parasite rate; SRTM: Shuttle Radar Topography Mission.

## Competing interests

The authors declare that they have no competing interests.

## Authors' contributions

AMN was responsible for study design, data cleaning, analysis, interpretation and production of the final manuscript. PWG contributed to the development of geostatistical models, analysis, interpretation and contributed to the final manuscript. VAA was responsible for geo-location of the survey data, data cleaning and preliminary analysis. APP contributed to the development of geostatistical models, analysis, interpretation and contributed to the final manuscript. SIH provided advice on analysis, interpretation of results and helped with the preparation of final manuscript. EM contributed to supervision of data collection and production of final manuscript. EJ contributed to supervision of data collection, provision of general policy framework, and production of final manuscript. RWS was responsible for overall scientific management, analysis, interpretation and preparation of the final manuscript. All authors have read and approved the final manuscript.

## Pre-publication history

The pre-publication history for this paper can be accessed here:

http://www.biomedcentral.com/1471-2334/9/180/prepub

## Supplementary Material

Additional file 1 **The relationship of ecological and climatic covariates with *Pf*PR_2-10_**. Detailed description of the ecological and climatic covariates and their relationship with *Pf*PR_2-10_. Click here for file

Additional file 2 **Bayesian model-based geostatistical modelling procedures**. Technical details of the Bayesian geostatistical models including prior specifications, implementation and output. Click here for file

## References

[B1] RBM The global malaria action plan 2008 Roll Back Malaria partnership. Geneva: World Health Organization

[B2] Hay SI Guerra CA Gething PW Patil AP Tatem AJ A world malaria map: *Plasmodium falciparum *endemicity in 2007 PLoS Med 2009 6 e1000048 19323591 10.1371/journal.pmed.1000048 PMC2659708

[B3] Butler RJ Atlas of Kenya: A comprehensive series of new and authenticated maps prepared from the national survey and other government sources with gazetteer and notes on pronunciation and spelling Nairobi, Kenya, the Survey of Kenya 1959

[B4] Craig MH Snow RW le Sueur D A climate-based distribution model of malaria transmission in sub-Saharan Africa Parasitol Today 1999 15 105 111 10.1016/S0169-4758(99)01396-4 10322323

[B5] Omumbo J Hay S Goetz S Snow R Rogers D Updating historical maps of malaria transmission intensity in East Africa using remote sensing Photogramm Eng and Remote Sens 2002 68 161 166 PMC3694357 23814324

[B6] Snow RW Gouws E Omumbo J Rapuoda B Craig MH Tanser FC le Sueur D Ouma Models to predict the intensity of *Plasmodium falciparum *transmission: applications to the burden of disease in Kenya T Roy Soc Trop Med H 1998 92 601 606 10.1016/S0035-9203(98)90781-7 10326100

[B7] Omumbo JA Hay SI Snow RW Tatem AJ Rogers DJ Modelling malaria risk in East Africa at high-spatial resolution Trop Med Int Health 2005 10 557 566 10.1111/j.1365-3156.2005.01424.x 15941419 PMC3191364

[B8] Snow RW Gilles HM Warrel DA, Gilles HM The epidemiology of malaria Bruce-Chwatt's essential malariology 2002 4 London: Arnold

[B9] Hay SI Smith DL Snow RW Measuring malaria endemicity from intense to interrupted transmission Lancet Infect Dis 2008 8 369 378 10.1016/S1473-3099(08)70069-0 18387849 PMC2653619

[B10] Hay SI Snow RW The Malaria Atlas Project: developing global maps of malaria risk PLoS Med 2006 3 e473 10.1371/journal.pmed.0030473 17147467 PMC1762059

[B11] Metselaar D van Thiel PH Classification of malaria Trop Geogr Med 1959 11 157 161

[B12] Smith DL McKenzie FE Snow RW Hay SI Revisiting the basic reproductive number for malaria and its implications for malaria control PLoS Biol 2007 5 e42 10.1371/journal.pbio.0050042 17311470 PMC1802755

[B13] Smith DL Dushoff J Snow RW Hay SI The entomological inoculation rate and *Plasmodium falciparum *infection in African children Nature 2005 438 492 495 10.1038/nature04024 16306991 PMC3128496

[B14] Smith DL Guerra CA Snow RW Hay SI Standardizing estimates of the *Plasmodium falciparum *parasite rate Malaria J 2007 6 131 10.1186/1475-2875-6-131 PMC2072953 17894879

[B15] Kleinschmidt I Bagayoko M Clarke GPY Craig MH Le Sueur D A spatial statistical approach to malaria mapping Int J Epidemiol 2000 29 355 361 10.1093/ije/29.2.355 10817136

[B16] Kleinschmidt I Omumbo JA Briët O Giesen N van de Sogoba N Mensah N Windmeijer P Moussa M Teuscher T An empirical malaria distribution map for West Africa Trop Med Int Health 2001 6 779 786 10.1046/j.1365-3156.2001.00790.x 11679126

[B17] Gemperli A Vounatsou P Sogoba N Smith T Malaria mapping using transmission models: application to survey data from Mali Am J Epidemiol 2006 163 289 297 10.1093/aje/kwj026 16357113

[B18] Guerra CA Hay SI Lucioparedes LS Gikandi PW Tatem AJ Assembling a global database of malaria parasite prevalence for the Malaria Atlas Project Malaria J 2007 6 17 10.1186/1475-2875-6-17 PMC1805762 17306022

[B19] Omumbo JA Snow RW *Plasmodium falciparum *parasite prevalence in East Africa: a review East Afr Med J 2004 81 649 656 15868982

[B20] PUBMED http://www.ncbi.nlm.nih.gov/pubmed/Accessed 7^th ^June 2009

[B21] Africa E-repositories http://www.connecting-africa.net Accessed 7^th ^May 2009

[B22] Ministry of Health Kenya Kenya National Malaria Indicator Survey 2007 2008 Nairobi, Kenya: Ministry of Health

[B23] UNICEF Vitamin E deficiency in Kenya: a report of a national micronutrient survey 1994 United Nations Children's Fund, Nairobi, Kenya

[B24] Mwaniki DL Omwega AM Muniu EM Mutunga JN Akelola R Shako BR Gotink MH Pertet AM The 1999 National Survey Report on Anaemia and the status of Iron, Vitamin A and Zinc in Kenya 1999 Ministry of Health, Nairobi, Kenya

[B25] Malaria Atlas Project http://www.map.ox.ac.uk/acknowledgements/Accessed 7^th ^June 2009

[B26] Ministry of Education Inception Report: Consultancy on development of a GIS database of learning Institutions (School mapping exercise), 2008 Oakar Services Ltd

[B27] Ministry of Roads and Public Works Classified Digital Road Network in Kenya Roads Department, Nairobi 2004

[B28] Alexandria Digital Library http://www.alexandria.ucsb.edu Accessed 15^th ^June 2009

[B29] Global Gazetteer Version 2.1 http://www.fallingrain.com/world/Accessed 17th June 2009

[B30] Pull JH Grab B Simple epidemiological model for evaluating malaria inoculation rate and risk of infection in infants Bull World Health Organ 1974 51 507 516 4549501 PMC2366319

[B31] Molineaux L Wernsdorfer W, McGregor I The epidemiology of human malaria as an explanation of its distribution, including some implications for its control. Malaria: Principles and Practice of Malariology 1988 2 London, Churchill Livingstone 913 998

[B32] Omumbo JA Guerra CA Hay SI Snow RW The influence of urbanisation on measures of *Plasmodium falciparum *infection prevalence in East Africa Acta Trop 2005 93 11 21 10.1016/j.actatropica.2004.08.010 15589793 PMC3191363

[B33] Hay SI Guerra CA Tatem AJ Atkinson PM Snow RW Urbanization, malaria transmission and disease burden in Africa Nat Rev Microbiol 2005 3 81 90 10.1038/nrmicro1069 15608702 PMC3130901

[B34] Craig MH Kleinschmidt I Nwan JB Le Seur D Sharo BL Exploring 30 years of malaria case data in KwaZulu-Natal, South Africa: Part I. The impact of climatic factors Trop Med Int Health 2004 9 1247 1257 10.1111/j.1365-3156.2004.01340.x 15598256

[B35] Craig MH Sharp BL Mabaso ML Kleinschmidt I Developing a spatial-statistical model and map of historical malaria prevalence in Botswana using a staged variable selection procedure Int J Health Geogr 2007 6 44 10.1186/1476-072X-6-44 17892584 PMC2082025

[B36] Kazembe LN Kleinschmidt I Holtz TH Sharp BL Spatial analysis and mapping of malaria risk in Malawi using point-referenced prevalence of infection data Int J Health Geogr 2006 5 41 10.1186/1476-072X-5-41 16987415 PMC1584224

[B37] Cox J Craig MH Le Sueur D Sharp B MARA/HIMAL technical report Mapping malaria risk in the highlands of Africa 1999 London School of Hygiene and Tropical medicine, UK and Medical Research Council, Durban South Africa

[B38] Thomson MC Connor SJ D'Alessandro U Rowlingson B Diggle P Cresswell M Greenwood B Predicting malaria infection in Gambian children from satellite data and bed net use surveys: the importance of spatial correlation in the interpretation of results Am J Trop Med Hyg 1999 61 2 8 10432046 10.4269/ajtmh.1999.61.2

[B39] Hay SI Snow RW Rogers DJ From predicting mosquito habitat to malaria seasons using remotely sensed data: practice, problems and perspectives Parasitol Today 1998 14 306 313 10.1016/S0169-4758(98)01285-X 17040796

[B40] Guerra CA Gikandi PW Tatem AJ Noor AM Smith DL The limits and intensity of *Plasmodium falciparum *transmission: implications for malaria control and elimination worldwide PLoS Med 2008 5 e38 10.1371/journal.pmed.0050038 18303939 PMC2253602

[B41] Kleinschmidt I Sharp BL Clarke GPY Curtis B Fraser C Use of Generalized Linear Mixed Models in the Spatial Analysis of Small-Area Malaria Incidence Rates in KwaZulu Natal, South Africa Int J Epidemiol 2001 153 1213 1221 10.1093/aje/153.12.1213 11415957

[B42] Hoek W Van Der Konradsen F Amerasinghe PH Perera D Piyaratne M Amerasinghe FP Towards a risk map of malaria for Sri Lanka: the importance of house location relative to vector breeding sites Int J Epidemiol 2003 32 280 285 10.1093/ije/dyg055 12714550

[B43] Clements A Lwambo N Blair L Nyandindi U Kaatano G Kinung'hi S Webster J Fenwick A Brooker S Bayesian spatial analysis and disease mapping: tools to enhance planning and implementation of a schistosomiasis control programme in Tanzania Trop Med Int Health 2006 11 490 503 10.1111/j.1365-3156.2006.01594.x 16553932 PMC2202922

[B44] Diggle P Moyeed R Rowlingson B Thompson M Childhood malaria in the Gambia: a case-study in model-based geostatistics Appl Stat 2002 51 493 506

[B45] Isaacs E Srivastava R Applied geostatistics 1989 Oxford University Press

[B46] Smith DL Hay SI Endemicity response timelines for *Plasmodium falciparum *elimination Malaria J 2009 8 87 10.1186/1475-2875-8-87 PMC2686731 19405974

[B47] Smith DL Noor AM Hay SI Snow RW Predicting changing malaria risk following expanded insecticide treated net coverage in Africa Trends Parasitol 25 11 511 6 10.1016/j.pt.2009.08.002 19744887 PMC2768685

[B48] Tatem AJ Noor AM von Hagen C di Gregorio A Hay SI High resolution population maps for low income nations: combining land cover and census in East Africa PLoS One 2007 2 e1298 10.1371/journal.pone.0001298 18074022 PMC2110897

[B49] Tatem AJ Noor AM Hay SI Defining approaches to settlement mapping for public health management in Kenya using medium spatial resolution satellite imagery Remote Sens Environ 2004 93 42 52 10.1016/j.rse.2004.06.014 PMC3350067 22581984

[B50] Central Bureau of Statistics 1999 Population and housing Census, Vol. 1: population distribution by administrative areas and urban Centers Nairobi Kenya 2001

[B51] Lysenko AJ Semashko Lebedew AW IN Geography of malaria. A medicogeographic profile of an ancient disease [in Russian] 1968 Itogi Nauki: Medicinskaja Geografija. Moscow: Academy of Sciences, USSR 25 146

[B52] Snow RW Omumbo JA Lowe B Molyneux CS Obiero J Palmer A Weber M Pinder M Nahlen B Obonyo C Newbold C Gupta S Marsh K Relation between severe malaria morbidity in children and level of *Plasmodium falciparum *transmission in Africa Lancet 1997 349 1650 1654 10.1016/S0140-6736(97)02038-2 9186382

[B53] Okiro EA Al-Taiar A Reyburn H Idro R Berkley J Nokes DJ Snow RW Age patterns of severe paediatric malaria and their relationship to *Plasmodium falciparum *transmission intensity Malaria J 2009 8 e4 10.1186/1475-2875-8-4 PMC2630996 19128453

[B54] Okiro EA Hay SI Gikandi PW Sharif SK Noor AM Peshu N Marsh K Snow RW The decline in paediatric malaria admissions on the coast of Kenya Malaria J 2008 6 151 10.1186/1475-2875-6-151 PMC2194691 18005422

[B55] O'Meara WP Bejon P Mwangi TW Okiro EA Snow RW Newton CJRC Marsh K Dramatic reductions in malaria morbidity and mortality are heralded by changes in age and presentation of disease Lancet 2008 372 1555 1562 10.1016/S0140-6736(08)61655-4 18984188 PMC2607008

[B56] Snow RW Marsh K The consequences of reducing transmission of *Plasmodium falciparum *in Africa Adv Parasitol 2002 52 235 264 full_text 12521262 10.1016/s0065-308x(02)52013-3

[B57] World Health Organization Malaria Rapid Test Diagnostic Performance 2008 Results of WHO product testing of malaria RDTs: Round 1 WHO, Geneva

[B58] Roberts L Enserink M Did they really say.eradication? Science 2007 318 1544 45 10.1126/science.318.5856.1544 18063766

[B59] Feachem R Sabot O A new global malaria eradication strategy Lancet 2008 371 1633 1635 10.1016/S0140-6736(08)60424-9 18374409

[B60] Greenwood B Fidock D Kyle D Kappe S Alonso P Collins F Duffy P Malaria: progress, perils, and prospects for eradication J Clin Invest 2008 118 1266 1276 10.1172/JCI33996 18382739 PMC2276780

[B61] Lines J Schapira A Smith T Tackling malaria today BMJ 2008 337 869 10.1136/bmj.a869 18713802

