# Supplementary material for: The risks of malariainfection in Kenya in 2009
Source: BMC Infect Dis. 2009 Nov 20;9:180. doi: 10.1186/1471-2334-9-180 (PMC2783030; doi:10.1186/1471-2334-9-180)
Supplement: Additional file 1 — The relationship of ecological and climatic covariates with PfPR2-10. Detailed description of the ecological and climatic covariates and their relationship with PfPR2-10. [file 1471-2334-9-180-S1.DOC]

**Additional File 1: The relationship of ecological and climatic covariates with *Pf*PR_2-10_**

A set of ecological and climatic covariates that have traditionally been used in malaria mapping were identified and assembled from a variety of sources. These covariates were then categorized into biologically plausible classes which were extracted at each survey location using ArcGIS 9.2 (ESRI Inc., USA). To assess the effects of the covariates on observed *Pf*PR_2-10_, a chi-square test of the difference in mean prevalence was undertaken. In addition, a univariate binomial logistic regression model was implemented for each covariate with *Pf*PR_2-10_ as the dependent variable in Stata/SE Version 10 (Stata Corporation, College Station, TX, USA). The results of the univariate analyses were used to determine an appropriate suite of covariates for inclusion in the Bayesian geostatistical model.

**1.1 Urban extents**

Urbanization has been shown to limit the availability of optimum environments for the development of the malaria transmitting anopheline populations resulting in reduced vector density, biting rates and transmission intensity in Kenya [1] and other African countries [2-7]. To define urban extents in Kenya the 1999 national census urban-rural definition of enumeration areas (EA) was used [8]. EA maps for 54 of 69 districts in Kenya were obtained from the Kenya National Bureau of Statistics to classify survey locations into urban or rural. For those 15 districts where data were not available, a combination of urban-rural defined point settlement data [9] and maps of sub-locations (administrative units above the EA) were used (Figure 1.1a). Where the urban-defined settlement point fell in a sub-location polygon whose area was less than 5 km^2^ the entire polygon was accepted as urban (used to define 9% of survey points). If the point fell in a sub-location polygon larger than 5 km^2^ then a polygon of 5 km^2^ within the sub-location boundary was generated to examine whether the survey point was within (urban) or outside (rural) this area (used to define 1% of survey points).

**Figure 1.1a** Map and box plot of urbanization (based on the Kenya 1999 census enumeration maps and settlements) against *Pf*PR_2-10_.

**
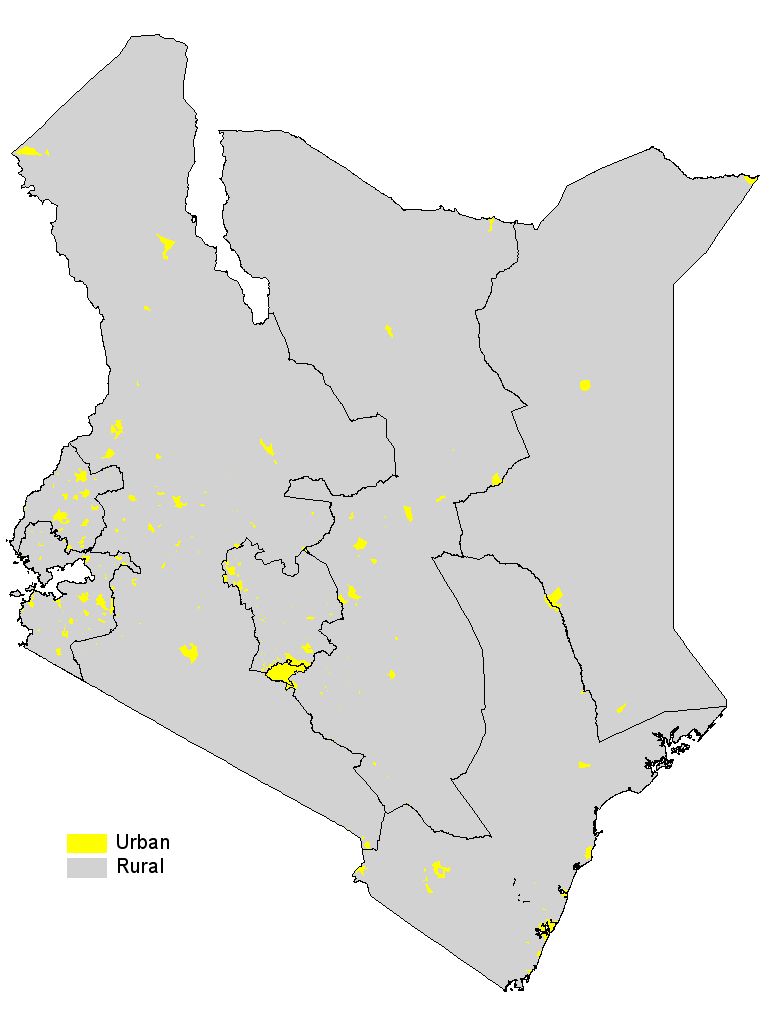
**

**Figure 1.1b** Box plots of *Pf*PR_2-10_ by urban-rural categories. The box indicates the inter-quartile range (25% and 75%); the black line within the box represents the median; and the whiskers represent the 2.5% and 97.5% centiles and outliers are plotted as circles outside this range.

**
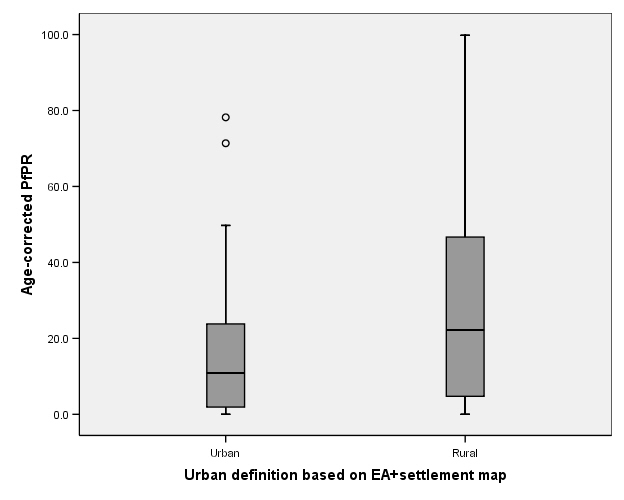
**

**Table 1.1:** Univariate analysis results of urbanization against *Pf*PR_2-10_

|  |  | |  | | |
| --- | --- | --- | --- | --- | --- |
|  | **Number of survey locations** | **Mean (median) PfPR_2-10_,**  **Chi^2^, P-value** | | **Univariate regression*:**  **Odds Ratio (95% CI), P-value** | **AIC values** |
|  |  |  | |  |  |
| **Urban (EA + settlement map)** |  |  | |  |  |
| Rural | 1,636 | 27.6 (21.9) | | Ref |  |
| Urban | 458 | 15.4 (11.9) | | 0.48 (0.34, 0.66), <0.001 | 0.87 |
|  |  | 3300.0, <0.001 | |  |  |

*In the univariate analysis here and for subsequent covariates the category most likely to have the highest median *Pf*PR_2-10_ is used as the reference class. Therefore the odds ratios are expected to be below 1.00.

**1.2 Maximum and minimum temperature**

Temperatures of between 25°C and 30°C are considered optimum for *P. falciparum* sporogony [10-12]. For *P. falciparum,* sporogonic development takes approximately 9 days at 30°C, 10 days at 25°C, 11 days at 24°C and 23 days at 20°C [13]. Below 16°C sporogony stops and above 35°C it slows down substantially or ceases [14].

Monthly average temperature raster surfaces at 1×1 km resolution were downloaded from the WorldClim website [15] from which annual averages were derived. These surfaces were produced from global weather station temperature records gathered from a variety of sources for the period 1950-2000 and interpolated using a thin-plate smoothing spline algorithm, with altitude as a covariate, to produce a continuous global surface [16]. For Kenya, average annual minimum temperature was classified into areas of <16°C and ≥16°C; while average annual maximum temperature was classified into <25°C; 25-30°C and >30°C.

**Figure 1.2a:** Maps of categories of average annual maximum and minimum temperature

**
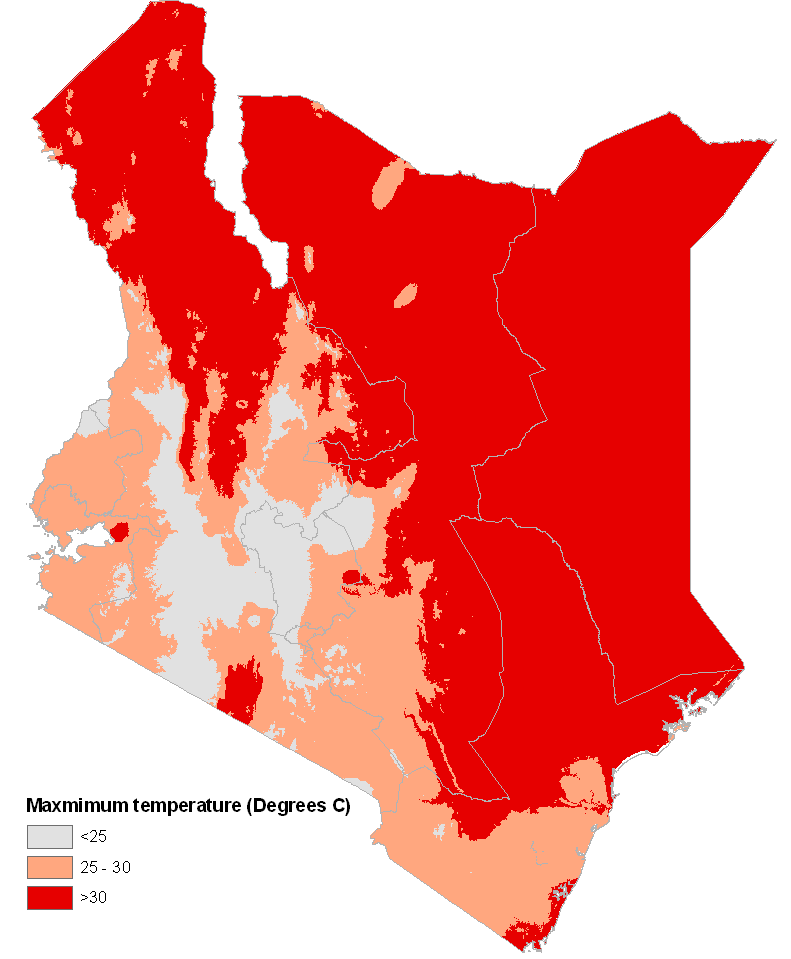
**
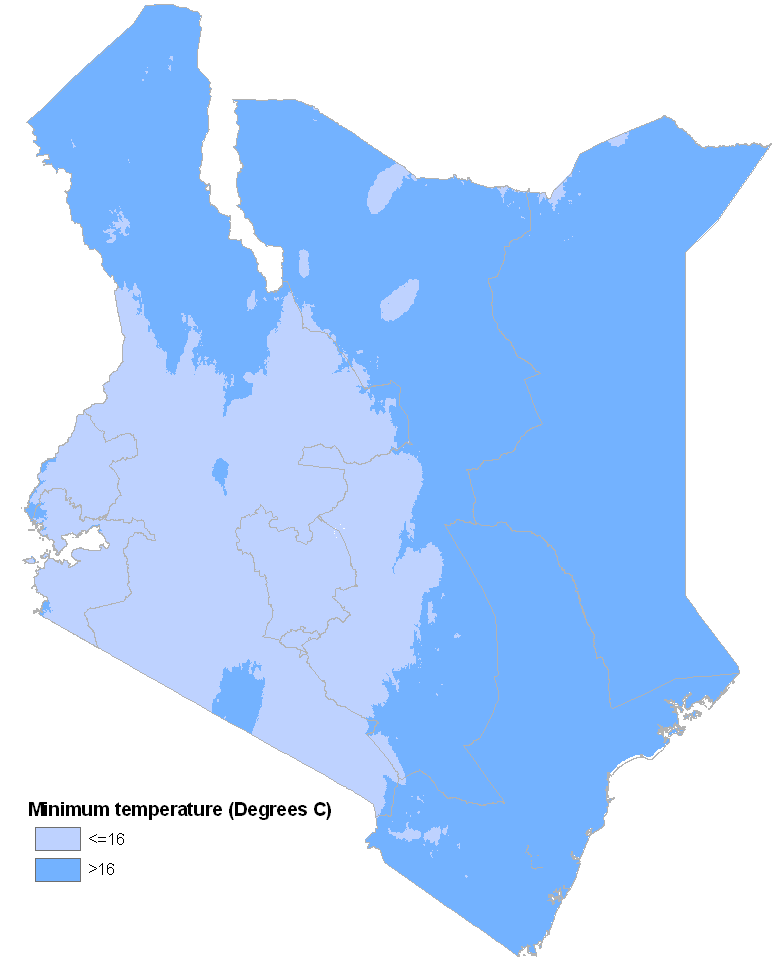


**Figure 1.2b:** Box plots of *Pf*PR_2-10_ by categories of average annual maximum and minimum temperature

**
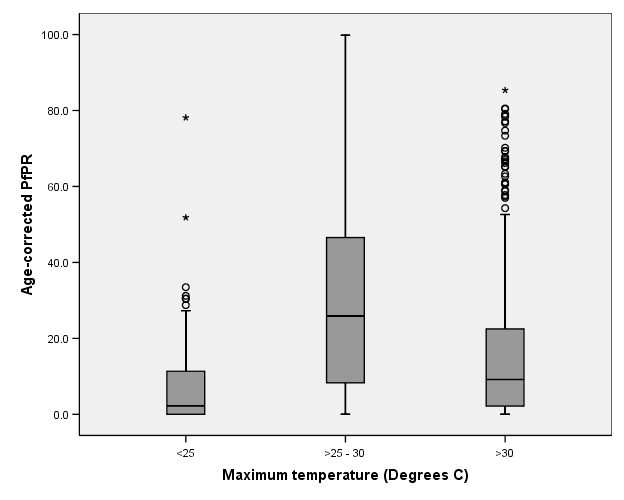
**
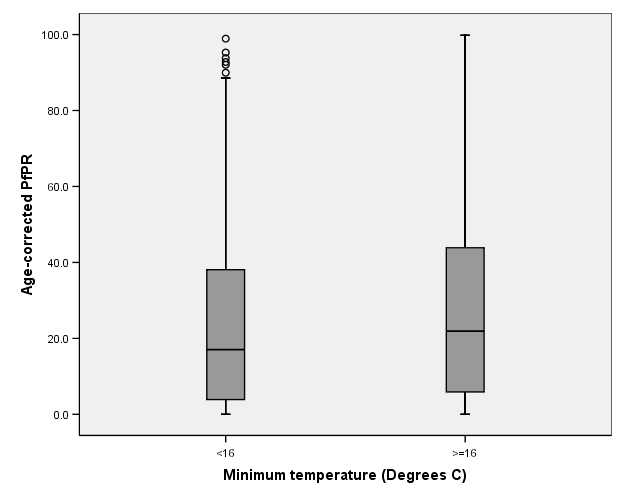


**Table 1.2: Univariate regression analysis results of maximum and minimum temperatures against *Pf*PR_2-10_**

|  |  | |  | |  | |
| --- | --- | --- | --- | --- | --- | --- |
|  | **Number of survey locations** | **Mean (median) PfPR_2-10_,**  **Chi^2^ , P-value** | | **Univariate regression*:**  **Odds Ratio (95% CI), P-value** | | **AIC values** |
|  |  |  | |  | |  |
| **Maximum temperature (Degrees Celsius)** |  |  | |  | |  |
| < 25 | 214 | 7.7 (2.3) | | 0.20 (0.10, 0.41), <0.001 | |  |
| 25-30 | 1628 | 29.8 (25.9) | | Ref | | 0.84 |
| >30 | 252 | 16.2 (9.2) | | 0.46 (0.35, 0.60), <0.001 | |  |
|  |  | 4700.0, <0.001 | |  | |  |
| **Minimum temperature (Degrees Celsius)** |  |  | |  | |  |
| <16 | 928 | 23.5 (17.1) | | 0.81 (0.66, 0.97), <0.036 | | 0.88 |
| ≥16 | 1166 | 27.6 (21.9) | | Ref | |  |
|  |  | 754.7, <0.001 | |  | |  |

**1.3 Precipitation**

Rainfall, combined with suitable ambient temperatures, provides potential breeding environments for *Anopheles* vectors while humidity is associated with vector longevity [17, 18]. Monthly mean precipitation raster surfaces at 1×1 km resolution were downloaded from the WorldClim website [15] and used as a proxy for rainfall. Different approaches have previously been adopted in malaria mapping for incorporating rainfall data into models in biologically appropriate ways for predicting malaria risk. These approaches include the direct use of continuous daily, monthly or annual mean rainfall [19, 20, 21]; the extraction of seasonal means from monthly mean rainfall data [22,23, 24]; the use of the mean rainfall of the lag month [25] or moving averages of three months computed from the mean of the survey month and the two preceding months [26]; and the use of categorical classes defined as areas of mean total annual rainfall of >80 mm [27]; the number of months in a year with rainfall >80 mm [11, 28] or >60 mm [23, 29]; or by using natural break points in the data [30].

For this study, areas with three continuous months of precipitation >60 mm and >80 mm in an average year were defined using mean monthly data (Figure 1.3a). In addition, the total annual precipitation was used to define areas of precipitation of 0-1000mm; 1001-1500mm; and >1500 mm annually to correspond respectively to arid and semi-arid, sub-humid and humid zones in Kenya [31].

**Figure 1.3a:** Maps of a) sets of three continuous months with precipitation >60 mm; b) sets of three continuous months with precipitation >80 mm; and c) areas of precipitation of 0-1000mm; 1001-1500mm; and >1500 mm.

**
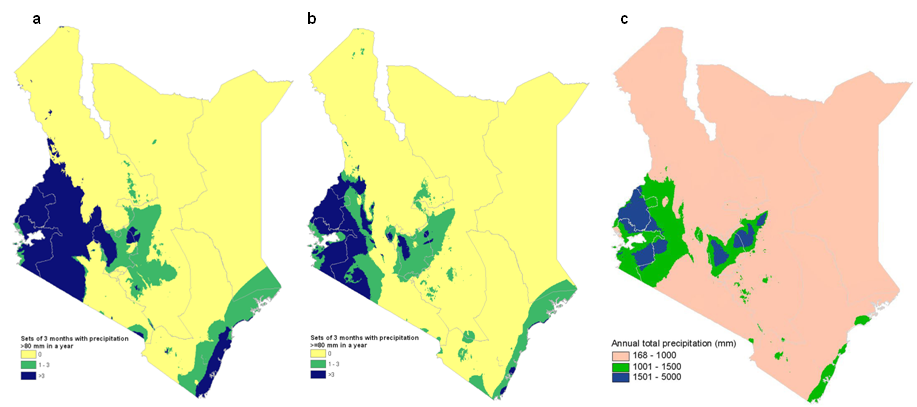
**

**Figure 1.3b:** Box plots of *Pf*PR_2-10_ by categories of sets of three continuous months in a year with precipitation >60 mm; sets of three continuous months in a year with precipitation >80; and areas of precipitation of 0-1000mm; 1001-1500mm; and >1500 mm.

**
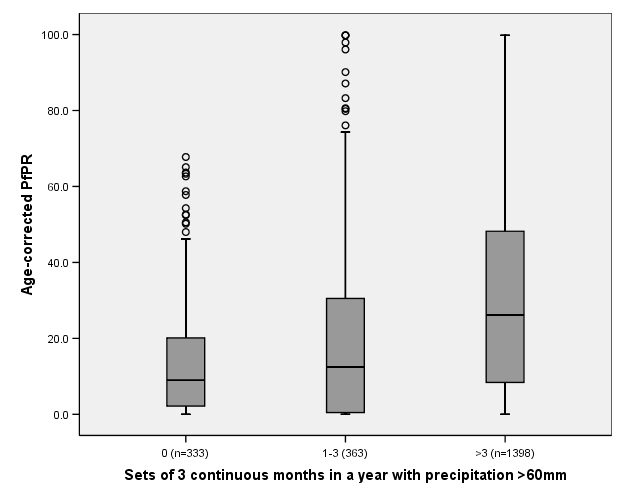

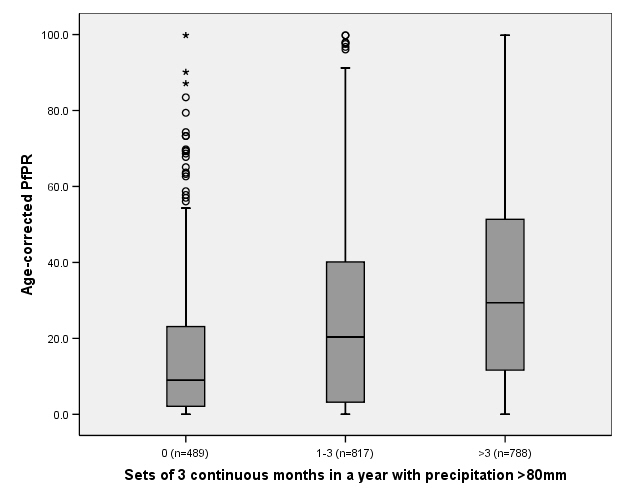
**

**
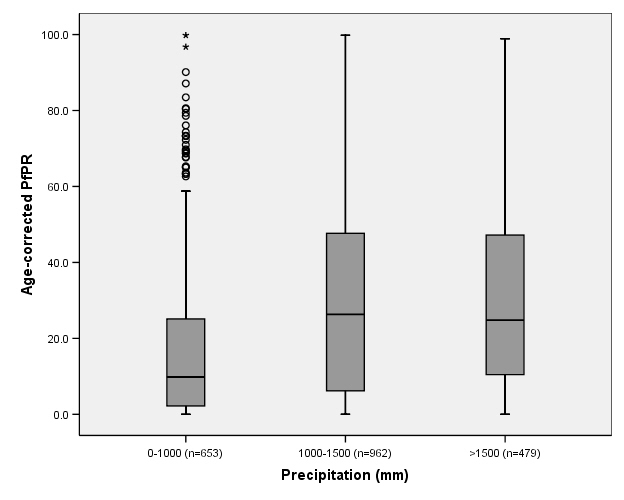
**

**Table 1.3:** Univariate analyses results of precipitation against *Pf*PR_2-10_

|  | **PfPR_2-10_** | | |  |
| --- | --- | --- | --- | --- |
|  | **Number of survey locations** | **Mean (median) PfPR_2-10_,**  **Chi^2^, P-value** | **Univariate regression*:**  **Odds Ratio (95% CI), P-value** | **AIC values** |
|  |  |  |  |  |
| **Sets of 3 consecutive months with precipitation >60 mm in a year** |  |  |  |  |
| 0 months | 1398 | 13.7 (9.0) | 0.37 (0.26, 0.51), <0.001 |  |
| 1-3 months | 333 | 19.6 (12.4) | 0.56 (0.42, 0.74), <0.001 | 0.84 |
| >3 months | 363 | 30.3 (26.1) | Ref |  |
|  |  | 8200.0, <0.001 |  |  |
| **Sets of 3 consecutive months with precipitation >80 mm in a year** |  |  |  |  |
| 0 | 788 | 15.3 (9.0) | 0.37 (0.28, 0.49), <0.001 | 0.86 |
| 1-3 | 489 | 25.0 (20.4) | 0.68 (0.54, 0.84), <0.001 |  |
| >3 | 817 | 33.0 (29.4) | Ref |  |
|  |  | 8100.0, <0.001 |  |  |
| **Annual mean total precipitation (mm)** |  |  |  |  |
| 0-500 | 479 | 16.5 (9.9) | 0.45 (0.34, 0.60), <0.001 |  |
| >1000-1500 | 653 | 29.8 (26.3) | 0.98 (0.77, 1.24), 0.850 | 0.88 |
| >1500 | 962 | 30.3 (24.8) | Ref |  |
|  |  | 7900.0, <0.001 |  |  |

**1.4 Aridity**

Enhanced vegetation index (EVI) and Normalized Difference Vegetation Index (NDVI) are both indices of intensity photosynthetic activity [32, 33]. Traditionally, NDVI has been used in malaria risk mapping as a proxy of rainfall [11, 19, 25, 27, 28, 29] and a measure of aridity that limits larval growth and vector survival [34]. EVI, just like NDVI, ranges from 0 (no vegetation) to 1 (complete vegetation), but is developed from satellite imagery of higher spatial and spectral resolution and corrects for some distortions in the reflected light caused by the particles in the air as well as the ground cover below the vegetation [35]. Monthly EVI surfaces have been derived from the global Moderate Resolution Imaging Spectroradiometer (MODIS) satellite imagery for the period 2001-2005 and subjected to temporal Fourier analysis at 1×1 km spatial resolution [33]. To define malaria-relevant EVI categories, threshold values were computed that corresponded to accepted definitions of aridity based on annual rainfall [31]. In Kenya this approximated to an EVI threshold of 0.3 (Figure 1.4a) below which average annual rainfall was less than 1000 mm corresponding to historical descriptions of malaria in Kenya referred to as “only malarious near water” [36].

**Figure 1.4a:**  Map of categories of enhanced vegetation index (EVI)

**
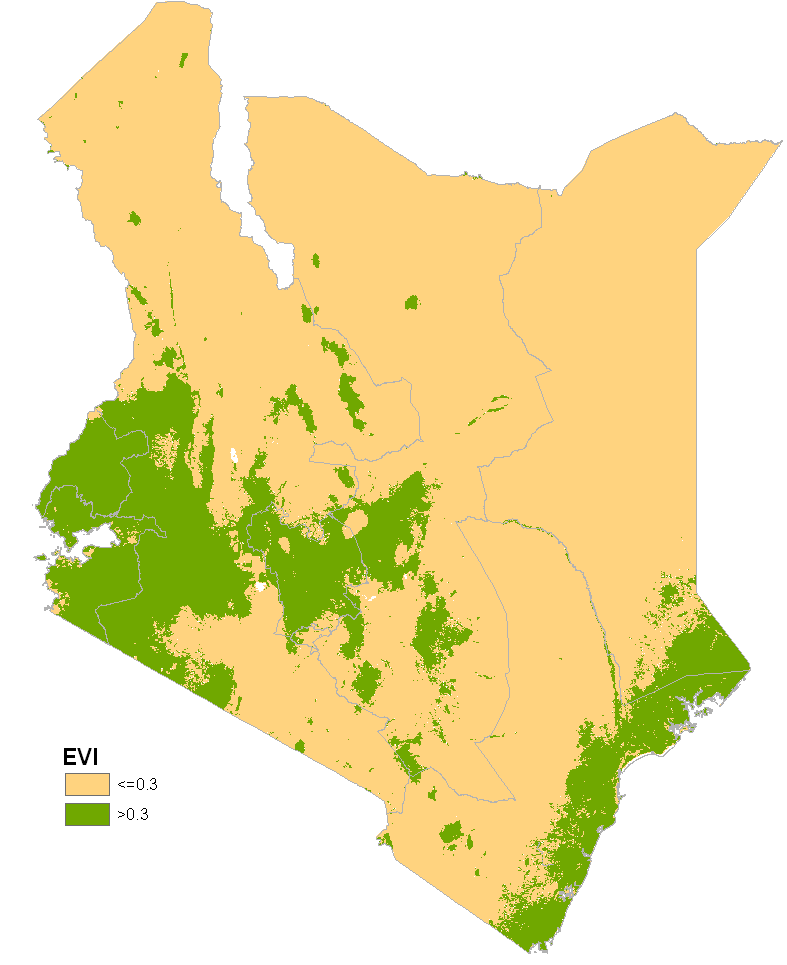
**

**Figure 1.4b:** Box plot of *Pf*PR_2-10_ by categories of EVI

**
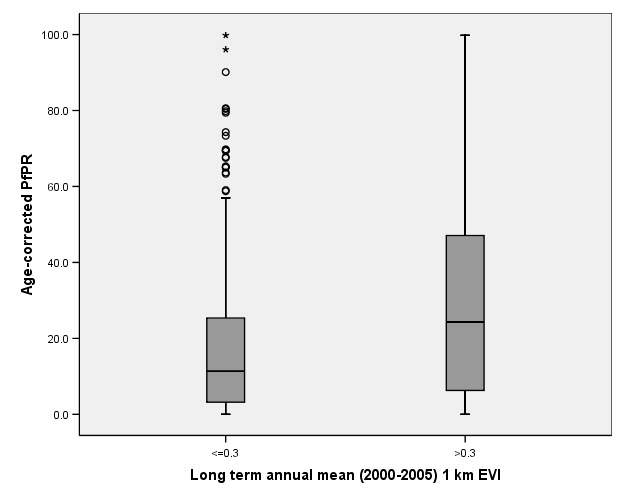
**

**Table 1.4** Univariate analyses results of EVI against *Pf*PR_2-10_

|  | **PfPR_2-10_** | | |  |
| --- | --- | --- | --- | --- |
|  | **Number of survey locations** | **Mean (median) PfPR_2-10_,**  **Chi^2^, P-value** | **Univariate regression*:**  **Odds Ratio (95% CI), P-value** | **AIC values** |
| **Categorical covariates**** |  |  |  |  |
| **Enhanced vegetation index** |  |  |  |  |
| > 0.3 | 1534 | 16.9 (11.3) | Ref |  |
| ≤ 0.3 | 560 | 29.0 (24.4) | 0.50 (0.39, 0.64), <0.001 | 0.87 |
|  |  | 3300.1, <0.001 |  |  |

**1.5: Altitude**

Altitude is inversely related with temperature and, in general, a reduction in temperature of up to 0.6°C is observed with every 100 m increase in altitude [22, 37]. We have elected to classify the biological relevance of altitude based on the coastal and Lake Victoria regions and the arid/semi-arid eastern lowlands (0-500 m above sea level); the savannah and the Rift Valley region (500-1500 m above sea level); and the central and western highlands (>1500 m above sea level) where these altitudinal limits impact on epidemic transmission [22]. An altitude map, at 30×30 m spatial resolution, was developed in 2008 from satellite imagery by Shuttle Radar Topography Mission (SRTM) project of the US National Geospatial-Intelligence Agency (NGA) and the National Aeronautical and Space Administration (NASA) and was downloaded from [38] (Figure 1.5a). Table 1.5 summarizes the results of the univariate regression analysis of altitude against *Pf*PR_2-10_.

**Figure 1.5a** Map *Pf*PR_2-10_ by altitude class

**
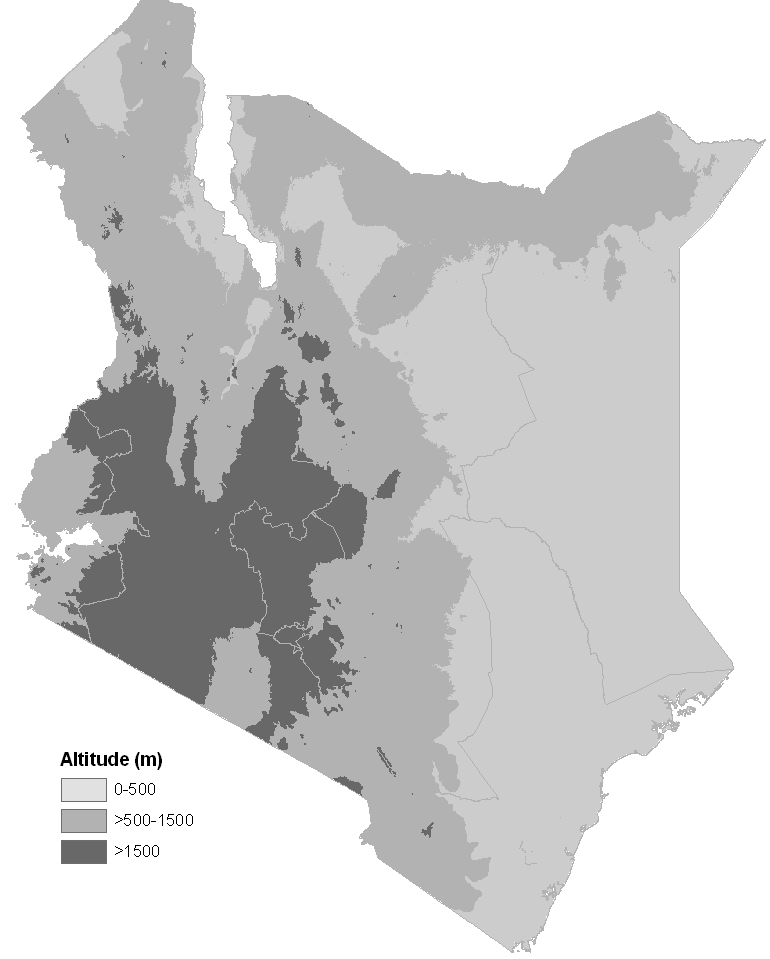
**

**Figure 1.5b** Box plot of *Pf*PR_2-10_ by categories of EVI

**
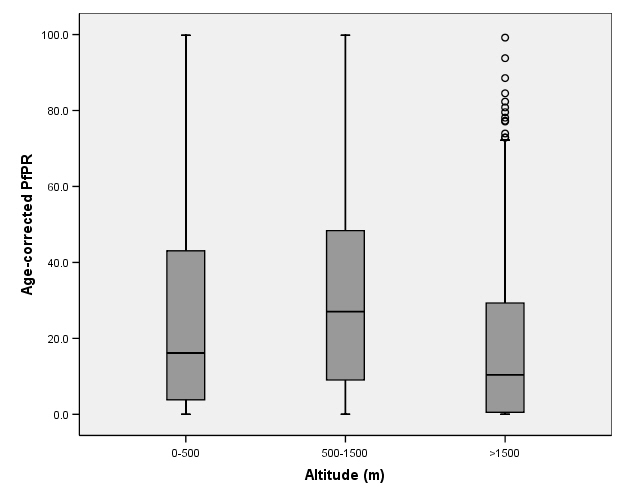
**

**Table 1.5:** Univariate analyses results of altitude against *Pf*PR_2-10_

|  | **PfPR_2-10_** | | |  |
| --- | --- | --- | --- | --- |
|  | **Number of survey locations** | **Mean (median) PfPR_2-10_,**  **Chi^2^, P-value** | **Univariate regression*:**  **Odds Ratio (95% CI), P-value** | **AIC values** |
| **Categorical covariates**** |  |  |  |  |
| **Altitude (m)** |  |  |  |  |
| 0 - 500 | 689 | 22.2 (13.0) | 0.59 (0.47, 0.74), <0.001 |  |
| >500 - 1500 | 860 | 32.6 (29.1) | Ref | 0.86 |
| >1500 | 545 | 19.4 (13.0) | 0.50 (0.39, 0.64), <0.001 |  |
|  |  | 4100.2, <0.001 |  |  |

**1.6 Perennial and seasonal water bodies**

Distance to permanent and temporary water bodies has previously been used in malaria mapping as a proxy for availability of potential breeding sites for the *Anopheles* vector [19, 21, 23, 24, 27, 30, 39]. A map of these water bodies for Kenya was created from a combination of two sources: a rivers layer digitized from 1:50,000 topographic maps and provided by the International Livestock Research Institute, and a map of water bodies developed by the Africover project [40]. Major perennial and seasonal water bodies were identified from the combined map by first excluding small and highly seasonal streams and tributaries (Figure 1.6a) and confirmed using Google Earth [41]. Euclidean distances (km) from these water bodies to the *Pf*PR survey locations were computed in ArcGIS 9.2 (ESRI Inc., USA) resulting in a 100×100 m distance surface (Figure 1.6a).

Previous studies have used distance to water bodies in malaria mapping in the continuous form [19, 21, 24, 27] or in the categorical form using natural break points [30] or other cut-offs [23]. Here, distance to water bodies was extracted at each survey location and the mean age-corrected parasite prevalence per kilometre was computed and plotted (Figure 1.6b). Three approaches were then used to inform the categorization of the distance to water bodies. First the survey locations were divided into those within and outside the median distance. Second, the mean distance was used as a cut-off. Third, the point of inflection on the plot (the distance at which parasite prevalence begins to decline) was visually determined and used as a cut-off. The box-plots of age-corrected parasite prevalence and the distance categories based on each of the three approaches were constructed (Figure 1.6c).

**Figure 1.6a** Map of main water features against and map of Euclidean distances to these water features

**
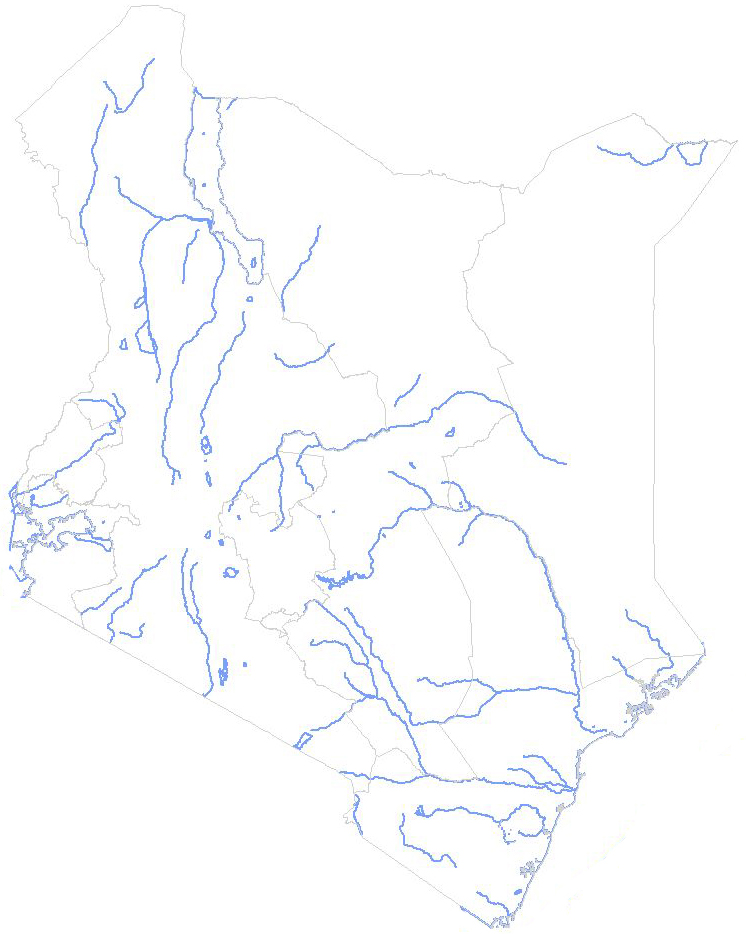

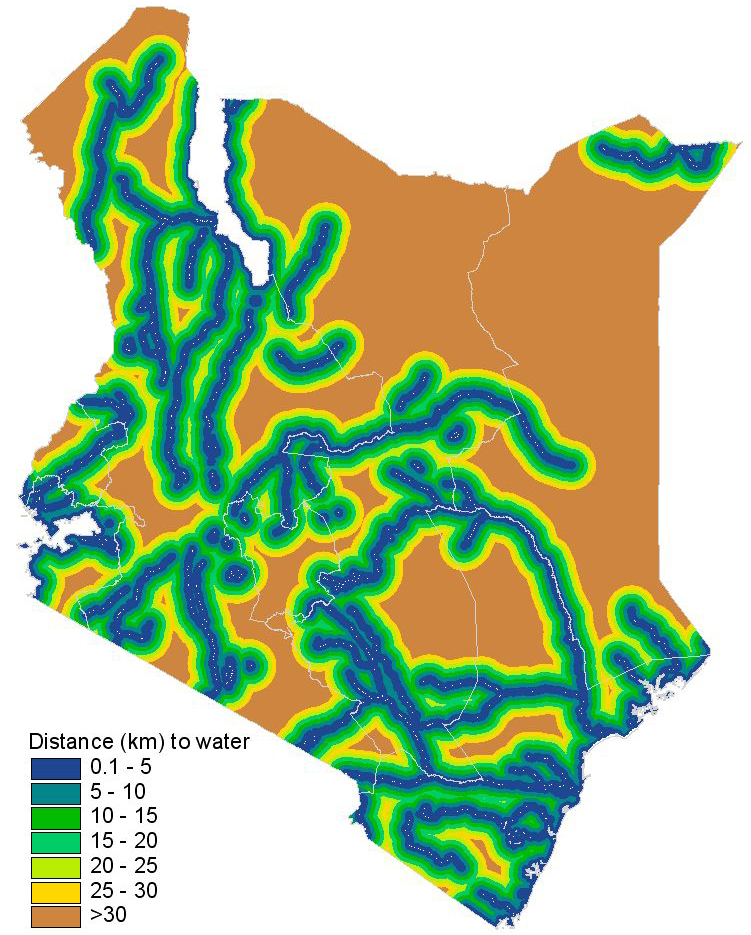
**

**Figure 1.6b:** Change of *Pf*PR_2-10_ with distance from water bodies. The red line shows the mean *Pf*PR_2-10_ per kilometre; the blue line shows a polynomial trend line fit to mean *Pf*PR_2-10_ data. Distance categories based on the mean (12 km); median (7 km) and the point of inflection (9km), the distance at which *Pf*PR_2-10_ begins to decline, were generated and their association with *Pf*PR_2-10_ examined.

**
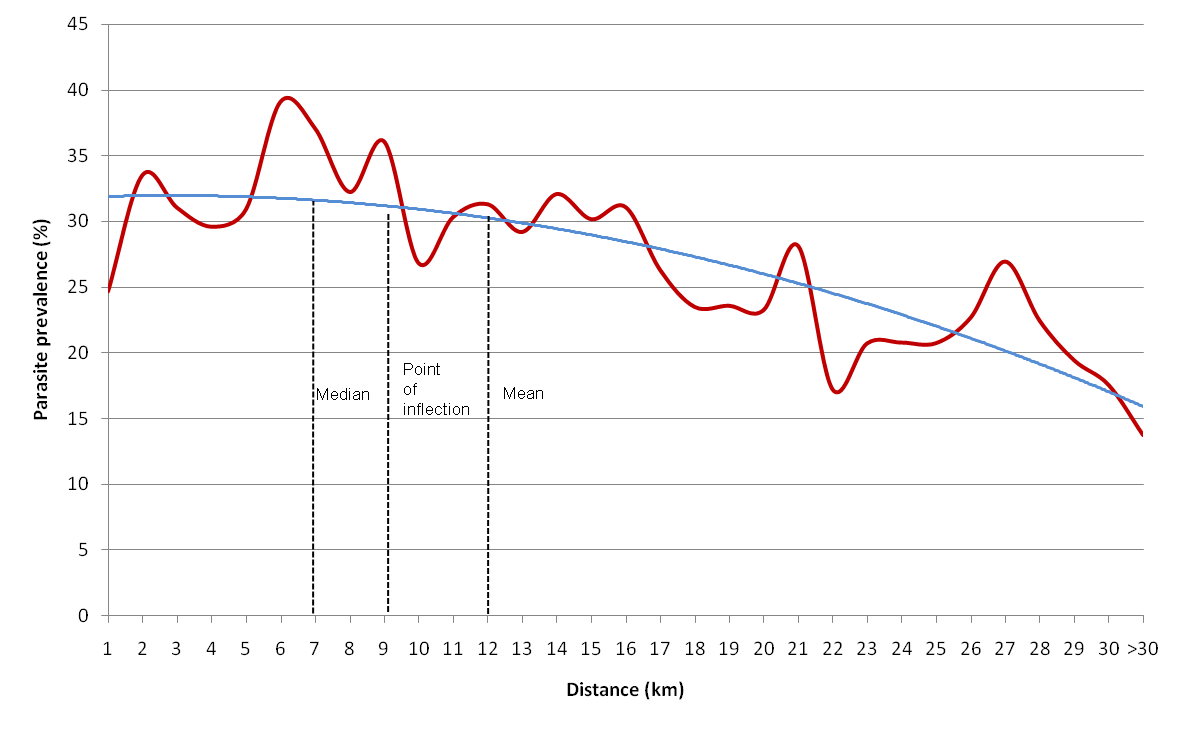
**

**Figure 1.6c:** Box plots of *Pf*PR_2-10_ by distance categories based on the median (7 km); mean (12 km) and the point of inflection at which *Pf*PR_2-10_ begins to decline (9 km).

**
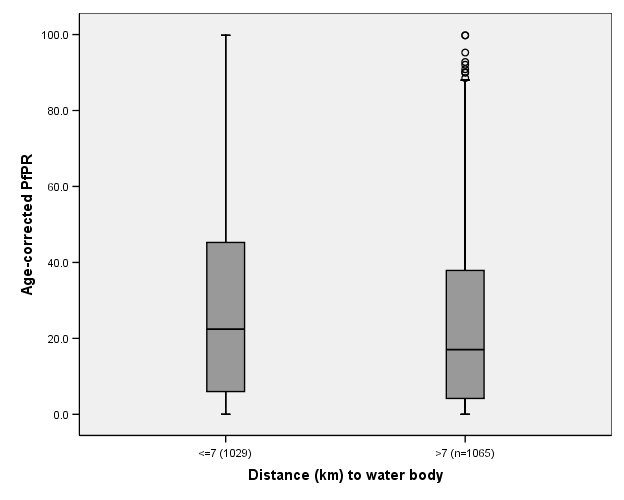

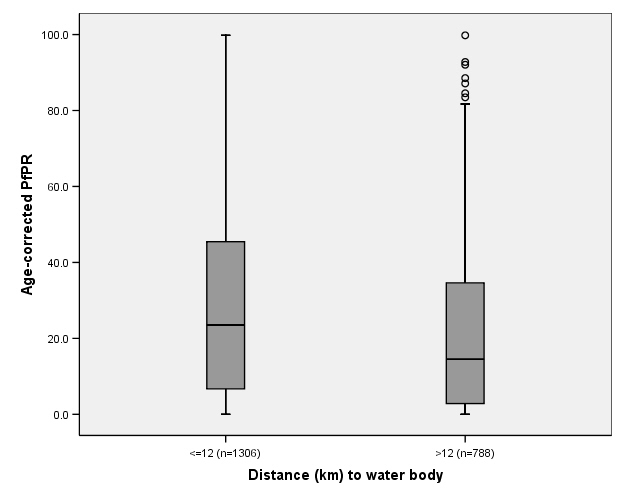
**

**
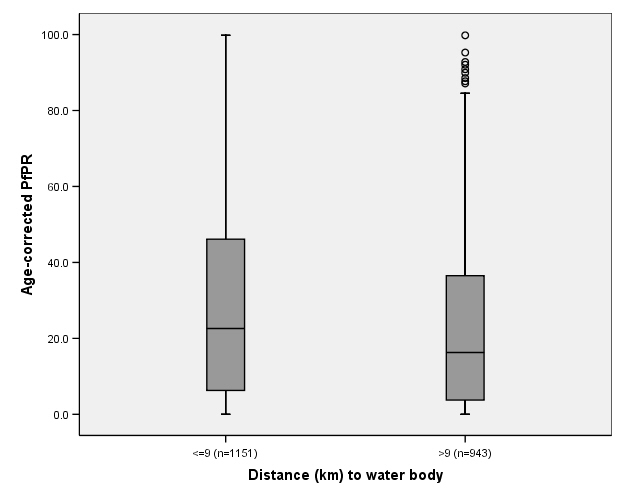
**

**Table 1.6:** Univariate analyses results of the three difference categories of distance to water bodies against *Pf*PR_2-10_

|  | **PfPR_2-10_** | | |  |
| --- | --- | --- | --- | --- |
|  | **Number of survey locations** | **Mean (median) PfPR_2-10_,**  **Chi^2^, P-value** | **Univariate regression*:**  **Odds Ratio (95% CI), P-value** | **AIC values** |
| **Categorical covariates**** |  |  |  |  |
| **Distance (km) to main water bodies** |  |  |  |  |
| ≤ 7 median distance | 1029 | 28.0 (22.4) | Ref | 0.88 |
| >7 median distance | 1065 | 23.6 (17.1) | 0.79 (0.65, 0.97), 0.022 |  |
|  |  | 1600, <0.001 |  |  |
| **Distance (km) to main water bodies** |  |  |  |  |
| ≤ 12 mean distance | 1306 | 28.6 (23.5) | Ref | 0.85 |
| >12 mean distance | 788 | 21.1 (14.5) | 0.67 (0.54, 0.82), <0.001 |  |
|  |  | 3300, <0.001 |  |  |
| **Distance (km) to main water bodies** |  |  |  |  |
| ≤ 9 distance at point of inflection | 1151 | 28.3 (22.6) | Ref | 0.88 |
| >9 distance at point of inflection | 943 | 22.7 (16.3) | 0.74 (0.61, 0.90), <0.004 |  |
|  |  | 2700, <0.001 |  |  |

**References**

1. Omumbo JA, Guerra CA, Hay SI, Snow RW: **The influence of urbanisation on measures of *Plasmodium falciparum* infection prevalence in East Africa**. *Acta Trop* 2005*,* **93**: 11-21.
2. Trape, JF, Zoulani A: **Malaria and urbanization in Central Africa: the example of Brazzaville. Part II: results of entomological surveys and epidemiological analysis**. *Trans R Soc Trop Med Hyg* 1987, **81**: 10–18.
3. Hay SI, Guerra CA, Tatem AJ, Atkinson PM, Snow RW: **Urbanization, malaria transmission and disease burden in Africa**. *Nat Rev Microbiol* 2005, **3**: 81-90.
4. Wang SJ, Lengeler C, Mtasiwa D, Mshana T, Manane L, Maro G, Tanner M: **Rapid Urban Malaria Appraisal (RUMA) II: epidemiology of urban malaria in Dar es Salaam (Tanzania)**. *Malaria J* 2006, **5**: 28.
5. Wang SJ, Lengeler C, Smith TA, Vounatsou P, Akogbeto M, Tanner M: **Rapid Urban Malaria Appraisal (RUMA) IV: epidemiology of urban malaria in Cotonou (Benin)**. *Malaria J* 2006, **5**: 45.
6. Wang SJ, Lengeler C, Smith TA, Vounatsou P, Cisse G Tanner M: **Rapid Urban Malaria Appraisal (RUMA) III: epidemiology of urban malaria in the municipality of Yopougon (Abidjan)**. *Malaria J* 2006*,* **5**: 29.
7. Wang SJ, Lengeler C, Smith TA, Vounatsou P, Diadie DA, Pritroipa X, Convelbo N, Kientga M, Tanner M: **Rapid urban malaria appraisal (RUMA) I: epidemiology of urban malaria in Ouagadougou**. *Malaria J* 2005, **4**: 43.
8. Central Bureau of Statistics: **1999 Population and housing Census, Vol. 1: population distribution by administrative areas and urban Centers**. Nairobi Kenya; 2001.
9. Ministry of Roads and Public Works: **Classified Digital Road Network in Kenya**. Roads Department, 2004, Nairobi.
10. Molineaux L: **The epidemiology of human malaria as an explanation of its distribution, including some implications for its control. Malaria: Principles and Practice of Malariology**. W.Wernsdorfer and I. McGregor. London, Churchill Livingstone. 2: 913-998, 1988.
11. Craig MH, Snow RW, le Sueur D: **A climate-based distribution model of malaria transmission in sub-Saharan Africa**. *Parasitol Today* 1999, **15**: 105–111.
12. Snow RW, Gilles HM: **The epidemiology of malaria. In: Warrel DA, Gilles HM editors. Bruce-Chwatt’s essential malariology**. 4^th^ ed. London: Arnold, 2002.
13. Boyd MF: **Epidemiology of malaria: factors related to the intermediate host**. In: Malariology. Philadelphia, Saunders: 551-607, 1959.
14. Detinova TS: **Age grouping methods in Diptera of medical importance, with special reference to some vectors of malaria**. Geneva, World Health Organization. 122-150, 1962.
15. WorldClim. <http://www.worldclim.org/download.htm>. Accessed 7th June 2009.
16. Hijmans R, Cameron S, Parra J, Jones P, Jarvis A: **Very high resolution interpolated climate surfaces for global land areas**. *Intl J Climatology* 2005, **25**: 1965-1978.
17. Dutta HM, Dutt AK: **Malaria ecology: a global perspective**. *Soc Sci Med* 1978, **12**: 69-84.
18. Gill CA: **The role of meteorology in malaria**. *Ind J Med Research* 1920, **8**: 633-693.
19. Omumbo JA, Hay SI, Snow RW, Tatem AJ, Rogers DJ: **Modelling malaria risk in East Africa at high-spatial resolution**. *Trop Med Int Health* 2005, **10**: 557-566.
20. Craig MH, Kleinschmidt I, Nwan JB, Le Seur D, Sharo BL: **Exploring 30 years of malaria case data in KwaZulu-Natal, South Africa: Part I. The impact of climatic factors**. *Trop Med Int Health* 2004, **9**: 1247-1257.
21. Noor AM, Clements ACA, Gething PW, Moloney G, Borle M, Shewshuk T, Hay SI, Snow RW: **Spatial prediction of *Plasmodium falciparum* prevalence in Somalia**. *Malaria J* 2008, **7**: e159
22. Cox J, Craig MH, Le Sueur D, Sharp B: **MARA/HIMAL technical report 1999: Mapping malaria risk in the highlands of Africa**. London School of Hygiene and Tropical medicine, UK and Medical Research Council, Durban South Africa, 1999.
23. Kleinschmidt I, Bagayoko M, Clarke GPY, Craig MH, Le Sueur D: **A spatial statistical approach to malaria mapping**. *Int J Epidemiol* 2000, **29**: 355-361.
24. Kleinschmidt I, Omumbo JA, Briët O, van de Giesen N, Sogoba N., Mensah N., Windmeijer P., Moussa M, Teuscher T: **An empirical malaria distribution map for West Africa**. *Trop Med Int Health* 2001, **6**: 779-786.
25. Hay SI, Snow RW, Rogers DJ: **From predicting mosquito habitat to malaria seasons using remotely sensed data: practice, problems and perspectives**. *Parasitol Today* 1998, **14**: 306–313.
26. Tanser FC, Sharp BL, Le Sueur D: **Potential effect of climate change on malaria transmission in Africa**. *Lancet* 2003, **362**: 1792-1798.
27. Gemperli A, Vounatsou P, Sogoba N, Smith T: **Malaria mapping using transmission models: application to survey data from Mali**. *Am J Epidemiol* 2006, **163**: 289-297.
28. Craig M H, Sharp B L, Mabaso M L, Kleinschmidt I: **Developing a spatial-statistical model and map of historical malaria prevalence in Botswana using a staged variable selection procedure**. *Int J Health Geogr* 2007, **6**: 44.
29. Snow RW, Gouws E, Omumbo J, Rapuoda B, Craig MH, Tanser FC, le Sueur D, Ouma: **Models to predict the intensity of *Plasmodium falciparum* transmission: applications to the burden of disease in Kenya**. *T Roy Soc Trop Med H* 1998*,* **92**: 601-606.
30. Kazembe LN, Kleinschmidt I, Holtz TH, Sharp BL: **Spatial analysis and mapping of malaria risk in Malawi using point-referenced prevalence of infection data**. *Int J Health Geogr* 2006, **5**:41.
31. Module 1: **An overview of cattle production systems in sub-Saharan Africa.** <http://www.ilri.org/InfoServ/Webpub/Fulldocs/ImprovingCattle/Module1.htm>. Accessed 17th June 2009.
32. Tucker CJ, Pinzon JE, Brown ME, Slayback DA, Pak EW; Mahoney R, Vermote EF, Saleous N: **An extended AVHRR 8-km NDVI dataset compatible with MODIS and SPOT vegetation NDVI data**. *Int J Remote Sens* 2005*,* **26**: 4485 – 4498.
33. Scharlemann JPW, Benz D, Hay SI, Purse BV, Tatem AJ: **Global data for ecology and epidemiology: a novel algorithm for temporal Fourier processing MODIS data**. *PLoS One* 2008, **3**: e1408.
34. Guerra CA, Gikandi PW, Tatem AJ, Noor AM, Smith DL, et al: **The limits and intensity of *Plasmodium falciparum* transmission: implications for malaria control and elimination worldwide**. *PLoS Med* 2008, **5**: e38.
35. NASA Earth Observatory: <http://earthobservatory.nasa.gov/Features/MeasuringVegetation/measuring_vegetation_4.php>. Accessed 17th June 2009.
36. Butler RJ: **Atlas of Kenya:** **A comprehensive series of new and authenticated maps prepared from the national survey and other government sources with gazetteer and notes on pronunciation and spelling**. Nairobi, Kenya, the Survey of Kenya, 1959.
37. Schwetz J: **Recherches sur la limite altimetrique du paludisme dans le Congo orientale et sur la cause de cette limite**. *Annales de la Société Belge de Médecin Tropicale* 1942, **22**: 183-208.
38. <http://glcf.umiacs.umd.edu/data/srtm/index.shtml>. Accessed 17th June 2009.
39. Van Der Hoek W., Konradsen F, Amerasinghe PH, Perera D, Piyaratne M, Amerasinghe FP: **Towards a risk map of malaria for Sri Lanka: the importance of house location relative to vector breeding sites**. *Int J Epidemiol* 2003*,* **32**: 280-285.
40. FAO Africover:[www.africover.org](http://www.africover.org/). Accessed 17th June 2009.
41. Google Earth: <http://earth.google.com/>. Accessed 17th June 2009.
