# Supplementary material for: The risks of malariainfection in Kenya in 2009
Source: BMC Infect Dis. 2009 Nov 20;9:180. doi: 10.1186/1471-2334-9-180 (PMC2783030; doi:10.1186/1471-2334-9-180)
Supplement: Additional file 2 — Bayesian model-based geostatistical modelling procedures. Technical details of the Bayesian geostatistical models including prior specifications, implementation and output. [file 1471-2334-9-180-S2.DOC]

**Additional File 2: Bayesian model-based geostatistical modelling procedures**

**2.1 Model overview**

The underlying value of *Plasmodium falciparum* parasite rate in the 2-10 year age cohort, , at each location for the year 2009 was modelled as a transformation of a spatiotemporally structured field superimposed with additional random variation [1]. The count of individuals tested as *P. falciparum* positive from the total sample of in each survey was modelled as a conditionally independent binomial variate given the unobserved underlying age-standardized *Pf*PR_2-10_ value [2]. The spatiotemporal component was represented by a stationary Gaussian process with mean and covariance . The unstructured component was represented as Gaussian with zero mean and variance . Both the inference and prediction stages were coded using Python (PyMC version 2.0) [3]

***Mean definition***

The mean component was modelled as a linear function of time, , and whether the prediction location was: urban (denoted by the indicator variable ) rather than rural; of maximum temperatures <25°C () or >30°C () compared to between 25°C- 30°C; of zero () or 1-3 () sets of three continuous months of precipitation >60 mm in average year compared to >3 sets; at distance to water bodies of >12 km () rather than ≤12 km; and of Enhanced Vegetation Index (EVI) of ≤0.3 () rather than >0.3. The mean component was therefore defined by nine parameters:

where denotes the intercept. Each survey was referenced temporally using the mid-point (in decimal years) between the recorded start and end months.

***Covariance definition***

Covariance between spatial and temporal locations was modelled using a spatially anisotropic space-time covariance function with a periodic component (wavelength=12 months) added to the time-marginal covariance model to capture seasonality [4]:

where is the modified Bessel function of the second kind of order , and is the gamma function [5, 6].

To allow for spatial anisotropy and to accommodate the effect of the curvature of the earth on point-to-point separations, spatial distance between a pair of points and was computed as great-circle distance multiplied by a factor that depends on the angle of inclination of the vector pointing from to . was computed as if latitude and longitude were Euclidean coordinates (on a cylindrical projection):

When (that is, for points at the same location but different times), the covariance function reduces to

As temporal separation increases, the covariance approaches a limiting sinusoid rather than zero. When , on the other hand (for points at different locations but the same time), it reduces to a standard exponential form with range parameter . Unlike standard sum-product models, this covariance function does not have problematic ridges along its axes [4].

**2.2 Table of prior specifications**

All the covariate coefficients had zero mean, standard deviation 100 normal priors. The covariate values were transformed by: (value - mean) / standard deviation.

| **Parameter** | **Prior** |
| --- | --- |
|  |  |
|  |  |
|  |  |
|  |  |
|  |  |
|  |  |
|  |  |

**2.3 Model implementation and output**

Bayesian inference was implemented using Markov Chain Monte Carlo to generate samples from the posterior distribution of the Gaussian field at each data location and of the unobserved parameters of the mean, covariance function and Gaussian random noise component.

Samples were generated from the mid-year 2009 mean of the posterior distribution of at each prediction location. For each sample of the joint posterior, predictions were made using space-time conditional simulation over the 12 months of 2009 [7]. These predictions were made at points on a regular 1 × 1 km spatial grid across Kenya. Model output therefore consisted of samples from the predicted posterior distribution of the 2009 annual mean *Pf*PR_2-10_ at each grid location, which were used to generate point estimates (Figure 3a in the main text) (computed as the mean of each set of posterior samples) and endemicity class membership probabilities (computed as the proportion of posterior samples for each pixel falling in the various class ranges). These latter values were used to present maps of both the class with the highest posterior probability of membership (Figure 3b in the main text), and the probability associated with that assignment (Figure 5 in the main text).

**References**

1. Hay SI, Guerra CA, Gething PW, Patil AP, Tatem AJ et al.: **A world malaria map: *Plasmodium falciparum* endemicity in 2007**. *PLoS Med* 2009, **6**: e1000048.
2. Diggle PJ, Thomson MC, Christensen OF, Rowlingson B, Obsomer V, et al: **Spatial modelling and the prediction of *Loa loa* risk: decision making under uncertainty**. *Ann Trop Med Parasitol* 2007, **101**: 499-509.
3. Anand Patil, David Huard and Christopher Fonnesbeck. PyMC 2.0: **Bayesian Stochastic Modelling in Python**. *J Stat Software* 2009, submitted.
4. Stein ML: **Space-time covariance functions**. *J Am Stat Assoc* 2005, **100**: 310-321.
5. Antosiewicz HA: **Bessel Functions of Integer Order. In: Abramowitz M, Stegun IA, editors. Handbook of Mathematical Functions: with Formulas, Graphs, and Mathematical Tables**. New York, U.S.A.: Dover Publications Inc. pp. 435-478, 1964.
6. Davis GM: **Gamma function and related functions. In: Abramowitz M, Stegun IA, editors. Handbook of Mathematical Functions: with Formulas, Graphs, and Mathematical Tables**. New York, U.S.A.: Dover Publications Inc. pp. 253-293, 1964.
7. Gething PW, Noor AM, Gikandi PW, Hay SI, Nixon MS, et al: **Developing geostatistical space-time models to predict outpatient treatment burdens from incomplete national data**. *Geogr Anal* 2008, **40**: 167-188.
